# Influence of Grassland Habitats on Acridoidea (Orthoptera) Species Diversity in Different Divisions of the Xinjiang Production and Construction Corps

**DOI:** 10.3390/biology14010014

**Published:** 2024-12-27

**Authors:** Yuxian Liu, Shaoshan Wang, Yuheng He, Guanzheng Yuan, Xingyu Pu, Chao Zhou

**Affiliations:** Xinjiang Production and Construction Corps Key Laboratory of Oasis Agricultural Pest Management and Plant Protection Resources Utilization, Xinjiang Uygur Autonomous Region, College of Agriculture, Shihezi University, Shihezi 832003, China; lyx18595671589@163.com (Y.L.); a13435617842@163.com (Y.H.); y18595671589@126.com (G.Y.); pusasa666@163.com (X.P.); zhou3441172087@163.com (C.Z.)

**Keywords:** Acridoidea, grassland type, species diversity, ecological environment

## Abstract

In this study, we investigated Acridoidea species and their populations in the grasslands managed by 13 divisions of the Xinjiang Production and Construction Corps (hereafter referred to as the Corps) during 2022–2024. We analyzed how the Corps’ grassland habitats influenced Acridoidea diversity. Using the netting method, we collected 5290 Acridoidea specimens, representing eight families, 37 genera, and 83 species. The Acridoidea were most abundant in mountain meadows and temperate grasslands, with 42 and 43 species, respectively, while the species richness in temperate desert grasslands and temperate steppe desert grasslands was comparably high. The highest diversity index of Acridoidea was recorded in temperate grasslands (3.053), followed by mountain meadows (2.563). Regarding altitude, the diversity index was highest in the 1000–1500 m range (3.237), followed by 500–1000 m (2.976) and 1500–2000 m (2.867). However, above 1500 m, the diversity index began to decline. The Acridoidea species diversity was significantly positively correlated with the average temperatures of the wettest and warmest seasons, as well as with precipitation levels during the wettest and driest months. The dominant grassland Acridoidea species were primarily distributed in areas with low-to-medium-low soil erodibility. These findings indicate that grassland types, elevation, temperature, humidity, and soil erodibility significantly influence Acridoidea habitats, survival, reproduction, and feeding behaviors, ultimately shaping their composition, distribution, and abundance across different habitats.

## Simple Summary

This study investigated Acridoidea (Orthoptera) species and grasshoppers in the grasslands managed by the Xinjiang Production and Construction Corps from 2022 to 2024. We collected 5290 specimens from eight families, 37 genera, and 83 species using the netting method. Acridoidea were most abundant in mountain meadows and temperate grasslands, with 42 and 43 species found, respectively. The diversity of Acridoidea was highest in temperate grasslands and mountain meadows. Diversity was also influenced by altitude, with the highest diversity found at elevations between 1000 and 1500 m. Factors such as temperature, precipitation, and soil erodibility were found to significantly affect Acridoidea habitats and populations. This study shows that grassland types, elevation, and environmental conditions play a key role in shaping locust distribution and abundance.

## 1. Introduction

Grasslands, as one of the predominant vegetation types in Xinjiang, cover a total area of 57.26 × 10⁶ hectares, representing 34% of the region’s total land area. In recent decades, Acridoidea damage in Xinjiang has escalated, with infestations affecting 8.5% of the available natural grassland area [1]. Among the 18 major grassland types identified in China, Xinjiang hosts 11, namely lowland meadows, temperate deserts, temperate steppe deserts, temperate desert grasslands, temperate steppes, temperate meadow grasslands, mountain meadows, alpine deserts, alpine grasslands, alpine meadows, and marshes [2].

The altitude in Xinjiang exhibits significant complexity, encompassing diverse landforms such as high mountains, basins, and deserts. In the northern region, including cities such as Urumqi and Shihezi, the elevation is relatively low, generally ranging from a few hundred to around one thousand meters. In contrast, the southern mountainous regions, such as the Pamir Plateau, have considerably higher elevations, averaging over 4500 m. Geographically located deep within Asia and far from the ocean, Xinjiang is surrounded by high mountains that block the influx of oceanic humid air currents, resulting in a hot and arid temperate continental climate. The average annual temperature in Xinjiang, influenced by geographic location, topography, and climatic factors, is approximately 7.6 °C [3]. Northern Xinjiang generally experiences lower annual temperatures compared to the region’s overall average, while southern Xinjiang has higher temperatures [4]. Xinjiang also features diverse soil types, although soil desertification is a prominent issue. The main soil types include brown desert soil, brown calcium soil, and gray-brown desert soil [5,6]. Variations in water and thermal resources across the region have given rise to diverse habitat types, which significantly influence the composition and diversity of Acridoidea communities [7].

Research on Acridoidea species diversity in Xinjiang began in the mid-1950s. With increasing focus on the distribution of Acridoidea in this region, new species have been continuously identified [8]. By 2012, records indicated 171 Acridoidea species belonging to eight families and 65 genera in Xinjiang [9]. Numerous studies have shown that the regional distribution of Acridoidea in Xinjiang exhibits distinct spatial and zonal patterns. Northern Xinjiang, particularly regions such as Altay, Tacheng, Boltara, Ili, and Changji, harbors a greater diversity and abundance of Acridoidea species compared to southern Xinjiang [10]. Conversely, southern Xinjiang is characterized by relatively fewer species. The distribution of Acridoidea species in Xinjiang is strongly influenced by ecological and geographical factors, including altitude, soil composition, and vegetation types [11]. Each zone displays unique characteristics in Acridoidea distribution patterns. Based on Xinjiang’s unique eco-geographical zones, topography, grassland resources, and vegetation characteristics, the grassland of this region can be classified into eight subzones. Each subzone supports distinct dominant Acridoidea species adapted to specific habitats, including variations in grassland type, vegetation, and altitude. Prolonged habitation within specific eco-geographical zones has led to consistent eco-physiological traits, behaviors, and morphological adaptations in different Acridoidea species. These adaptations further validate the dynamic interaction between Acridoidea and their habitats [12,13,14]. However, research reports on a wide variety of insects are very limited [15,16]; especially, studies on the relationship between Orthoptera Acridoidea and environmental factors in Xinjiang have not been reported.

In this study, we identified the species of Acridoidea and clarified the dominant species in each division of the Corps. The analysis of Acridoidea species diversity in the grasslands managed by the Corps was carried out using various methods, and the correlations between Acridoidea diversity and grassland type and altitude were examined. The influence of the soil erodibility factor on the distribution of the dominant species of grassland Acridoidea and the influence of ecological factors (temperature and rainfall) on species diversity were also investigated.

## 2. Materials and Methods

### 2.1. Survey Methodology

#### 2.1.1. Survey Methodology and Study Period

This study primarily employed the treadmill survey method, which is a technique often used in ecological and environmental studies, particularly when conducting surveys or assessments of species diversity or behavior in a specific area. It typically involves systematically walking through a study area while recording observations, such as species sightings, behaviors, or environmental conditions, over a defined route or transect. This was conducted June–August for three consecutive years, 2022–2024, in accordance with the biological characteristics of Acridoidea occurrence (the period of Acridoidea outbreak).

#### 2.1.2. Setting Up Investigation Sites for Trekking

In accordance with the 2021 National Grassland Pest Census Implementation Plan (printed version), routes for transect walks and investigation sites were planned based on the life and habitat of the types of Acridoidea within the Corps’ jurisdiction, which correspond to the respective regions or autonomous regions. The planning also considered factors such as grassland type, elevation, basins, mountains, plains, and other landforms, as well as the distribution of grasslands and townships.

The area of each investigation site was at least 0.5 ha, and the distance between adjacent investigation sites was no less than 1 km. In general, for local grasslands with an area of less than 200,000 ha, the area designated for trekking must be at least 3% of the total grassland area. For grasslands ranging from 200,000 ha to 540,000 ha, the trekking area must comprise at least 2.5% of the total area. For grasslands exceeding 540,000 ha, the trekking area must account for at least 2% of the total area.

In this study, a total of 157 routes and 992 investigation sites were established. Multiple routes were implemented within each division of the Corps, and typical Acridoidea occurrence zones were selected as investigation sites. These investigation sites were reassessed over a three-year period.

#### 2.1.3. Description of Study Site

**Figure 1:**
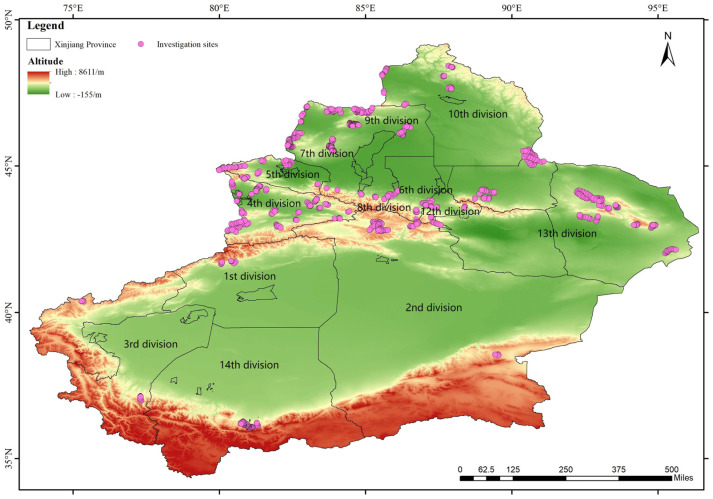
The 992 investigation sites in the 13 divisions of the Xinjiang Corps.

This study investigated grasslands within the jurisdiction of 13 divisions of the Xinjiang Corps, including Alar city, the 1st division, which is located in the Aksu region on Xinjiang’s southern border; Tiemenguan city, the 2nd division, which is located in the Bayinguoleng Mongol Autonomous Prefecture on the southern border; Tumushuke city, the 3rd division, which is located in the Kashgar region on the southern border; Kokdara city, the 4th division, which is located in the Ili Kazakh Autonomous Prefecture on the northern border; Shuanghe city, the 5th division, which is located in the Bortala Mongol Autonomous Prefecture on the northern border; Wujiaqu city, the 6th division, which is located north of Urumqi in the Changji Hui Autonomous Region in northern Xinjiang; Huyanghe city, the 7th division, which is located in Karamay and the Tacheng area of the Ili Kazakh Autonomous Prefecture in northern Xinjiang; Shihezi city, the 8th division; Xiaobaiyang city, the 9th division, which is located in the Tacheng area of the Ili Kazakh Autonomous Prefecture in northern Xinjiang; Beitun city, the 10th division, which is located in the Altay region of the Ili Kazakh Autonomous Prefecture in northern Xinjiang; the 12th division, which is located in the vicinity of Urumqi in the Changji Hui Autonomous Region in northern Xinjiang; Hongxing city, the 13th division, which is located in the Hami region of eastern Xinjiang; and Kunyu city, the 14th division, which is located in the Hotan region of southern Xinjiang (Figure 1).

This study investigated the grasslands within the jurisdiction of the 13 divisions of the Xinjiang Corps, covering an area of approximately 29,058,000 km^2^, which constitutes only 5% of the total grassland area of Xinjiang (572,588,000 km^2^). The distribution of the divisional farms and grassland profiles in the surveyed area is presented in Table 1.

### 2.2. Collection Methods

This study followed the “National Grassland Pest Census Implementation Program” and employed the standard plot sampling method for investigation. Five plots were selected within each investigation site, with each plot covering an area of approximately 25 m^2^. Acridoidea specimens were collected using a trap net in the upper vegetation layer, with the hand-held net (30 × 30 cm) swept once to the left and once to the right per pass. A total of 10 sweeps were performed per plot, resulting in 50 sweeps per standard investigation site.

Each investigation site was georeferenced using a mobile GPS device or other software, which recorded the latitude, longitude, elevation, habitat characteristics, and the identity of the collector.

The specimens collected from each plot were placed in 20 × 30 cm self-sealing bags containing 75% ethanol, with approximately 20 Acridoidea specimens (adults) per bag. Each bag was labeled with a collection tag, which included the specimen label number, collection location, date of collection, collector’s name, and other relevant information. The specimens were then transported to the laboratory for the identification of species, enumeration of Acridoidea, and recording of other pertinent data.

### 2.3. Specimen Preparation and Identification

Pin insertion method: Based on the size of the Acridoidea body, the appropriate type of insect pin was selected. The specimens were collected and soaked in 75% ethanol during field collection, and once the bodies had softened after drying, a pin was inserted in each specimen. The specimens were then preserved for future analysis, communication, and identification.

Dipping method: The Acridoidea specimens collected from the field and soaked in 75% ethanol were classified and identified according to their species using the line sample method. Specimens of the same species were then placed in the same 50 mL centrifuge tube, with approximately 20 heads per tube. The tubes were filled with 75% ethanol and stored for long-term preservation and subsequent statistical analysis.

The specimens collected from the field and brought to the laboratory were examined for morphological characteristics under a stereomicroscope. Classification and identification were performed with reference to authoritative works, including the *Taxonomy of Locusts* [17], *Zoologia of China: Insecta* [18], *Chinese Locust Research* [19], *Identification of Locusts in Xinjiang* [20], and other relevant texts. In classification studies of Chinese Acridoidea, scientists classified them into eight families: Oedipodidae [21], Arcypteridae [22], Gomphoceridae [23,24], Acrididae [25], Catantopidae [26], Pamphagidae [27], Chrotogonidae [28], and Pyrgomorphidae [29]. Specimens that were difficult to identify were sent to experts for help.

### 2.4. Data Processing

In this study, the classification of dominant Acridoidea species was based on the criteria established by Hu Jing et al. Specifically, a species is considered dominant when the number of individuals accounts for more than 10% of the total Acridoidea population. Species with a percentage between 1% and 10% are classified as common, while those with a percentage less than 1% are classified as rare [30].

The species dominance and evenness indices and the similarity coefficient of the collected locusts were analyzed using *Shannon–Wiener* index [31], *Pielou* index [32], and Jaccard similarity coefficient, respectively, following the methodology outlined in the *Principles and Methods of Biodiversity Research* [33]. The formulae for these two indices are as follows:

(1) $$\mathrm{Shannon}\text{–}\mathrm{Wiener}\;\mathrm{Index}:\;H=-\sum_{i=1}^{s}pi\ln pi$$

*Pielou* Index: *E* = *H*/*H*max, *H*max = ln*S* (2)

where *H*—diversity index, *S*—total number of species, *pi*—proportion of the individuals of the *i*th species in relation to total number of individuals in the specimen. The data were analyzed and processed using Excel version 2021and SPSS version 27.01.1.

The similarity between two grassland types was assessed using the *Jaccard* similarity coefficient [34]. The degree of similarity was categorized as follows: extremely dissimilar when the coefficient was less than 0.25, moderately dissimilar when it ranged from 0.25 to 0.50, moderately similar when it ranged from 0.50 to 0.75, and extremely similar when it exceeded 0.75. The formula for the index is as follows:

*Jaccard* similarity coefficient: *Cj* = *j*/[(*a* + *b*) − *j*] (3)

where *a*—number of Acridoidea species in grassland type A, *b*—number of Acridoidea species in grassland type B, *j*—number of Acridoidea species shared between grassland types A and B.

The environmental data for this study were sourced from WorldClim (https://www.worldclim.org/current/, accessed on 23 August 2024), and climatic data for each region of Xinjiang were extracted over a 30-year period using ArcGIS 10.8 [35]. The dataset included 19 biometeorological factors, with a spatial resolution of 2.5 arc-min. Pearson correlation analysis was performed, and correlation heat maps were generated by combining the climatic data for the 19 factors across the 13 divisions of the Corps in Xinjiang.

Soil-related data for this study were obtained from the Harmonized World Soil Database (HWSD), publicly available through the Food and Agriculture Organization (FAO) of the United Nations (https://www.fao.org/soils-portal/data-hub/soil-maps-and-databases/harmonized-world-soil-database-v12/en/, accessed on 20 August 2024). ArcGIS 10.8 was used to extract soil data for Xinjiang, including sand content, silt content, clay content, and organic carbon content.

In this study, we employed the method of the *EPIC* (Erosion Productivity Impact Calculator) model to calculate the soil erodibility factor (*K*) [36]. The classification of soil erodibility based on *K* values is as follows: slight erodibility when *K* ≤ 0.020, low erodibility when 0.020 < *K* ≤ 0.026, medium-low erodibility when 0.026 < *K* ≤ 0.033, medium erodibility when 0.033 < *K* ≤ 0.040, medium-high erodibility when 0.040 < *K* ≤ 0.046, and high erodibility when *K* > 0.046. A higher *K* value indicates an increased risk of soil erosion in the area.

(4) $$\begin{array}{l} K_{EPIC}=0.2+0.3\exp[-0.0256SAN*(1-SIL/100)]*\\ {\left(\frac{SIL}{CLA+SIL}\right)}^{0.3}*[1-\frac{0.25C}{C+\exp(3.72-2.95C)}]*\\ \left[1-\frac{0.7(1-SAN/100)}{\left(1-SAN/100)+\exp(-5.51+22.9(1-SAN/100)\right)}\right] \end{array}$$

(5) $$K=(-0.01383+0.51575K_{EPIC})*0.1317$$

Here, *SAN*—sand content (0.05–2.00 mm) (%); *SIL*—silt content (0.002–0.05 mm) (%); *CLA*—clay content (<0.002 mm) (%); *C*—organic carbon content (%). The resulting *KEPIC* values were subsequently converted to the international system of *K* values, expressed in units of t·ha·h/(MJ·mm·ha).

ArcGIS 10.8 was used to calculate the *K* values of soil erodibility in Xinjiang. Spatial distribution maps of the dominant grassland Acridoidea species using ArcGIS 10.8, based on the soil erodibility *K* values, were generated by combining data on the dominant Acridoidea species in each division of the Xinjiang Corps.

## 3. Results

### 3.1. Survey of Grassland Acridoidea Species in Each Division

In this study, a total of 5290 Acridoidea specimens were collected between June 2022 and August 2024. A total of 83 species from eight families and 37 genera were identified. These included 1 species in 1 genus of Pyrgomorphidae, 2 species in 2 genera of Pamphagidae, 1 species in 1 genus of Chrotogonidae, 6 species in 3 genera of Catantopidae, 29 species in 12 genera of Oedipodidae, 36 species in 11 genera of Arcypteridae, 5 species in 4 genera of Gomphoceridae, and 3 genera containing 3 species of Acrididae (species names are listed in Supplementary Materials). The dominant species in each division are as follows in Table 2.

### 3.2. Comparison of Grassland Acridoidea Species Diversity

As shown in Table 3, the *Shannon–Wiener* and *Pielou* indices of grassland Acridoidea varied across the divisions of the Corps. The *Shannon–Wiener* diversity index was highest in the 5th and 4th divisions, followed by the 9th division. The *Pielou* index was greatest in the 13th division, followed by the 14th and 10th divisions, while the 4th division exhibited the lowest *Pielou* index. These results indicate that the 5th and 4th divisions had the richest Acridoidea species diversity, whereas the 13th division demonstrated the most uniform distribution of Acridoidea populations across species.

### 3.3. Distribution of Grassland Acridoidea in Different Grassland Types

Acridoidea from the eight families exhibited varied patterns across grassland types. Among these, Acridoidea of the family Arcypteridae were the most widely distributed, occurring in all eight grassland types, with the highest number of species found in the mountain meadow and temperate grassland types. Oedipodidae species were present in seven grassland types but were absent from the temperate grassland type, with the highest number of species observed in the temperate desert class. Pyrgomorphidae species were restricted to the lowland meadow type, while those of the family Chrotogonidae were found exclusively in the temperate desert type (Table 4).

The total number of Acridoidea genera and species varied across grassland types. The lowland meadow, mountain meadow, temperate steppe, and temperate desert types exhibited higher distributions, with 16 genera and 28 species, 18 genera and 42 species, 22 genera and 43 species, and 16 genera and 38 species, respectively (Table 4).

The temperate desert grassland type supported the highest number of Acridoidea families (6). The second highest number of Acridoidea families (5) was distributed across lowland meadows, alpine meadows, mountain meadows, and temperate steppes. In contrast, three Acridoidea families were found in the temperate desert steppe and temperate steppe desert types. Only one family, Reticulitidae, was present in the temperate meadow grassland type.

**Figure 2:**
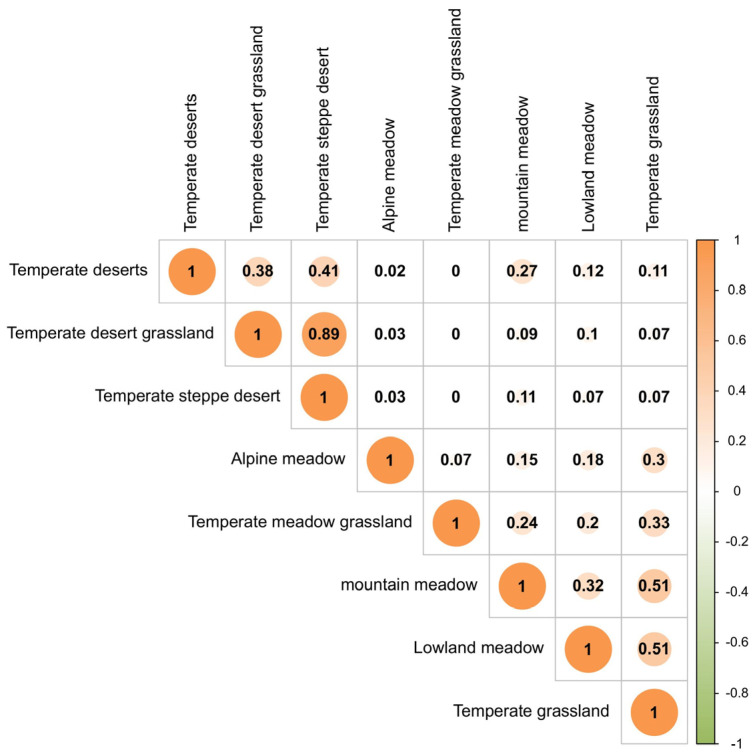
The *Jaccard* similarity coefficients of Acridoidea communities in different grassland types.

The *Jaccard* similarity coefficient between the temperate desert grassland and temperate steppe desert types was the highest (Figure 2). This indicates that these two grassland types shared a high number of identical Acridoidea species, exhibiting extremely similar distributions. The numbers of Acridoidea families, genera, and species in the temperate desert grassland and temperate steppe desert types were 3, 8, and 17, and 3, 7, and 17, respectively. In contrast, the similarity coefficients of the temperate grassland type with both the mountain meadow and lowland meadow types were 0.51, reflecting moderate similarity in species composition. The numbers of Acridoidea families, genera, and species were 5, 22, and 43, respectively, while the corresponding values were 5, 18, and 42 for the mountain meadow type and 5, 16, and 28 for the lowland meadow type. The *Jaccard* similarity coefficients of the temperate desert, temperate desert grassland, and temperate steppe desert types with the temperate meadow grassland type were all 0, and their similarity coefficients with the alpine meadow type were 0.03. This indicates that these grassland types share virtually no overlapping Acridoidea species distributions.

### 3.4. Relationship Between Grassland Acridoidea Species Diversity and Grassland Types

Table 5 presents the *Shannon–Wiener* and *Pielou* indices for Acridoidea across different grassland types. The temperate desert type recorded the highest number of Acridoidea specimens (1527), followed by the temperate grassland (1357) and mountain meadow (1257) types. Acridoidea were less abundant in the alpine meadow (86) and temperate meadow grassland (53) types. The *Shannon–Wiener* index was highest in the temperate grassland type (3.053), relatively low in the temperate desert grassland (1.887) and temperate steppe desert (1.703) types, and intermediate in the other grassland types. Among the eight grassland types, the alpine meadow and temperate grassland types exhibited the highest *Pielou* indices with values of 0.837 and 0.812, respectively. These findings suggest that grassland Acridoidea species are more diverse and evenly distributed in temperate grassland and mountain meadow types.

### 3.5. The Relationship Between Dominant Grassland Acridoidea Species and Soil Erodibility (K) Values

The *K* values of soil erodibility in Xinjiang were calculated to range from 0.020 to 0.088 based on the sand, silt, clay, and organic carbon content from regional soil data. The highest *K* values were observed in the Junggar Basin and Tarim Basin regions, classifying these areas as high-erodibility zones. Similarly, the eastern region of Xinjiang, parts of Altay, the areas between southern Tian Shan and the Tarim Basin, and the northern Kunlun Mountains exhibited higher *K* values, also falling within high-erodibility zones. In contrast, parts of the Yili Valley, Tacheng, and areas surrounding Wujiaqu, Shihezi, Karamay, and Huyanghe showed the lowest *K* values, indicating low-to-medium-low erodibility. The remaining regions, with *K* values ranging from approximately 0.035 to 0.088, were categorized as medium-to-high-erodibility zones.

**Figure 3:**
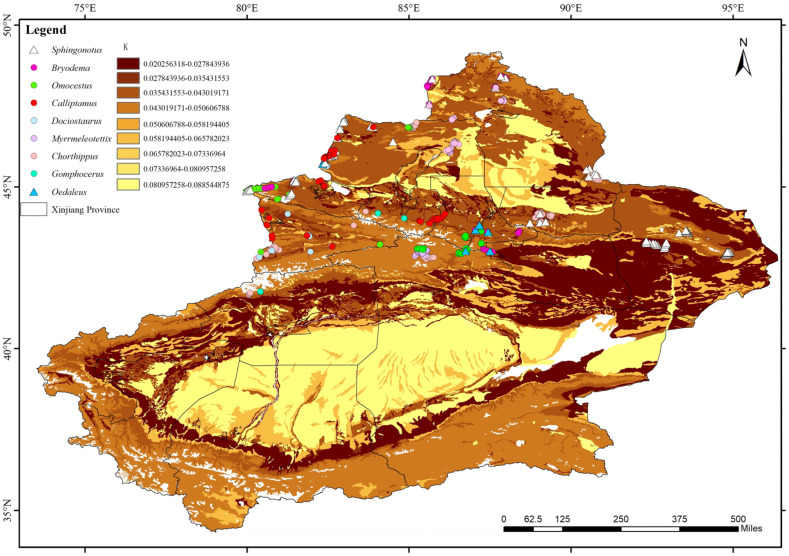
Soil erodibility in Xinjiang and the spatial distribution of dominant Acridoidea species in grasslands of various regions.

As illustrated in Figure 3, the majority of dominant Acridoidea species in each division of the Corps are primarily distributed in areas classified as low-erodibility or medium-low-erodibility. In contrast, their presence in regions with medium--to-high erodibility intervals is minimal or nonexistent.

### 3.6. The Relationship Between Grassland Acridoidea Species Diversity and Altitude

The *Shannon–Wiener* and *Pielou* indices for Acridoidea species in different altitude zones are shown in Table 6.

Table 6 indicates that the *Shannon–Wiener* index of Acridoidea species was highest in the altitude range of 1000–1500 m, with a value of 3.237. This was followed by the 500–1000 m and 1500–2000 m intervals, which had diversity indices of 2.976 and 2.867, respectively. These results suggest that the species diversity index of grassland Acridoidea increases with altitudes up to 1500 m, but declines at higher elevations.

The *Pielou* index of Acridoidea species was higher in the interval of 3000–3500 m at 0.837, compared to other altitude intervals, and it was lowest in the interval of 500–1500 m at 0.735.

The 1000–1500 m altitude range contained the highest number of grassland Acridoidea specimens, totaling 1831. This was followed by the 500–1000 m and 1500–2000 m altitude ranges, which recorded 1368 and 917 specimens, respectively. In contrast, the fewest Acridoidea specimens were observed in the 3000–3500 m altitude range, with only 86 individuals.

In terms of Acridoidea families, Catantopidae, Oedipodidae, Arcypteridae, and Gomphoceridae exhibited broader vertical distribution, occurring across the 0–3500 m altitude range. These families were predominantly concentrated within the 500–1500 m altitude interval. The family Pyrgomorphidae, however, was restricted to lower altitudes, whereas Arcypteridae represented the family with the highest species distribution at higher altitudes.

**Figure 4:**
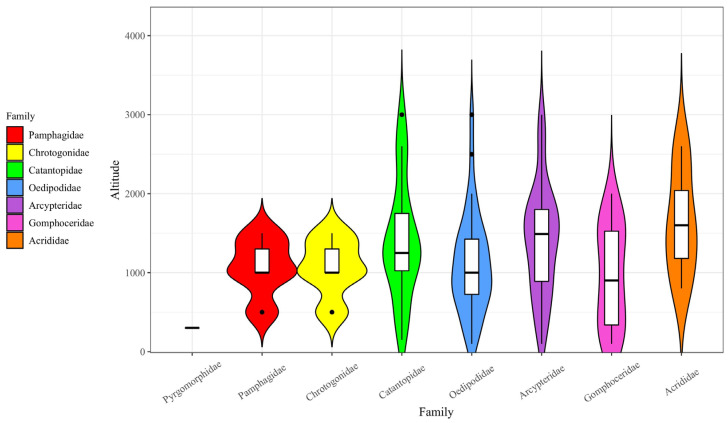
The proportion of species distribution of eight families of Acridoidea at different altitudes.

As shown in the proportions of species distribution across the eight families of Acridoidea at various altitudes (Figure 4), the number of Acridoidea species reached its peak at an altitude of 500–1500 m in most families. The vertical distributions of the Pamphagidae, Pyrgomorphidae, and Chrotogonidae families were notably narrower compared to the other families.

### 3.7. The Relationships of Acridoidea Species Diversity with Temperature and Rainfall

**Figure 5:**
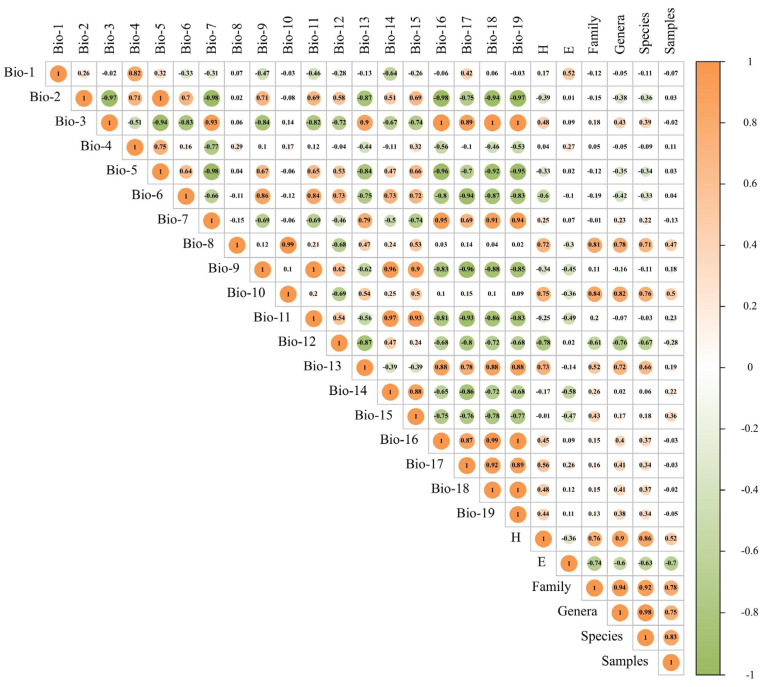
Pearson correlation analysis of species diversity of Acridoidea and environmental factors (Bio1–Bio19). H: Shannon–Wiener index; E: Pielou evenness index; Bio-1: annual average temperature/°C; Bio-2: annual average diurnal temperature range/°C; Bio-3: isothermal characteristic [(Bio-2/Bio-7) × 100]; Bio-4: temperature seasonality (standard deviation × 100); Bio-5: maximum temperature of the hottest month/°C; Bio-6: minimum temperature of the coldest month/°C; Bio-7: annual range of temperature (Bio-5 – Bio-6)/°C; Bio-8: average temperature of the wettest quarter/°C; Bio-9: average temperature of the driest quarter/°C; Bio-10: average temperature of the hottest quarter/°C; Bio-11: average temperature of the coldest quarter/°C; Bio-12: annual precipitation/mm; Bio-13: precipitation in the wettest month/mm; Bio-14: precipitation in the driest month/mm; Bio-15: precipitation seasonality (coefficient of variation); Bio-16: wettest quarterly precipitation/mm; Bio-17: driest quarterly precipitation/mm; Bio-18: precipitation in the hottest month/mm; Bio-19: precipitation in the coldest month/mm.

As shown in Figure 5, the diversity indices of grassland Acridoidea species in each division of the Corps exhibited a significant positive correlation (*p* > 0.05) with the average temperature of the wettest quarter (Bio-8), the average temperature of the warmest quarter (Bio-10), the precipitation level in the wettest month (Bio-13), and the precipitation level in the driest quarter (Bio-17). Furthermore, they showed a weak positive correlation (*p* < 0.05) with isothermality (Bio-3), the precipitation level during the wettest season (Bio-16), the precipitation level in the hottest quarter (Bio-18), and the precipitation level in the coldest quarter (Bio-19). A significant negative correlation was observed with the minimum temperature of the coldest month (Bio-6) and annual precipitation (Bio-12).

The *Pielou* index of grassland Acridoidea species in all divisions of the Corps was significantly and positively correlated with the annual average temperature (Bio-1). It was significantly negatively correlated with precipitation in the driest month (Bio-14), and weakly negatively correlated with the average temperature of the driest quarter (Bio-9), the average temperature of the coldest quarter (Bio-11), and precipitation seasonality (Bio-15).

The numbers of families, genera, and species of grassland Acridoidea in all divisions of the Corps were significantly positively correlated with the average temperature of the wettest season (Bio-8), the average temperature of the warmest season (Bio-10), and the precipitation level of the wettest month (Bio-13), and significantly negatively correlated with annual precipitation (Bio-12). They were also significantly positively correlated with the *Shannon–Wiener* index and significantly negatively correlated with the *Pielou* index. The specimen number of grassland Acridoidea in each division of the Corps was significantly positively correlated with the average temperature of the hottest quarter (Bio-10), the average temperature of the wettest quarter (Bio-8), and seasonal precipitation (Bio-15), and had no significant correlation with other ecological factors.

## 4. Discussion

### 4.1. Comparison of Acridoidea Species Diversity Across Corps Divisions

The comparison of grassland Acridoidea diversity indices, along with species number and evenness, revealed that the distribution density of Acridoidea individuals, as well as the distribution area and range of species, is strongly influenced by geographical conditions and the natural environment.

In comparison, the 4th, 5th, and 9th divisions exhibited a higher grassland Acridoidea diversity index and richer species than other divisions of the Corps. The 9th division is located in the Junggar Basin, with a lower average elevation, and has all eight grassland types investigated in this study, while the 5th and 4th divisions have seven grassland types, with habitats that provide favorable environments for more Acridoidea species to survive.

The grassland Acridoidea diversity indices of the 5th and 4th divisions were both high, at 2.979 and 2.883, respectively. However, the evenness indices differed, with the Ili Kazakh Autonomous Prefecture exhibiting the lowest evenness index (0.704) among the regions of Xinjiang. This indicates a highly uneven distribution of Acridoidea individuals across species, primarily due to the dominance of *Dociostaurus* (*Kazakia*) *brevicollis* in the specimen population. This dominance can be attributed to the significant proportion of specimens from *Dociostaurus* (*Kazakia*) *brevicollis* in these divisions. This further suggests that the dominant Acridoidea species are better adapted to each region’s ecological environment and exhibit higher competitive ability within the community.

The Acridoidea assemblages from all regions of the Xinjiang Corps exhibited high evenness (0.944 and 0.925) in the 13th and 14th divisions, indicating a more balanced distribution of individuals across all Acridoidea species in these divisions.

Considering the geographical and ecological characteristics of Xinjiang, it is evident that the natural environment in the northern foothills of the Tianshan Mountains (northern Xinjiang) is considerably more favorable than that in the southern region. The climate and soil conditions in the north are conducive to vegetation growth, with extensive areas cultivated for crops, thus creating a more suitable habitat for Acridoidea. Consequently, the 4th, 5th, 6th, 8th, and 12th divisions, as well as the 7th, 9th, and 10th divisions in northern Xinjiang, exhibited higher levels of overall species diversity compared to the southern divisions. This finding aligns with the report by Zhao Ling et al. [9], which indicates that Acridoidea species are more diverse and widely distributed in northern Xinjiang, particularly in Altay, Tacheng, Bortala, Yili, and Changji, than in the southern regions.

In this study, a total of 83 species of locust (belong to eight families and 37 genera) were identified, which constitutes less than 50% of the 65 genera and 171 species reported by Zhao L. [9]. This discrepancy may be attributed to several factors. Firstly, the development of pastoralism has led to increased efforts in controlling and preventing grassland locusts, potentially resulting in a reduction in Acridoidea species. Secondly, this study was limited to a locust survey within the grasslands in the Corps, covering an area of approximately 29,058,000 km^2^. This area represents only a small fraction of the total grassland area in Xinjiang, which spans 572,588,000 km^2^ [37]; thus, this contributed to the relatively small number of locust species observed. Thirdly, the soils in Xinjiang are primarily wind-sand and saline soils, which are increasingly vulnerable to erosion due to persistent wind and sand erosion, as well as other forms of degradation [38]. In an ecological environment characterized by deteriorating soil conditions and escalating soil erosion, the diversity of grassland Acridoidea species is likely to diminish, further explaining the lower number of Acridoidea species identified in this study compared to previous investigations.

### 4.2. Comparison of the Distribution and Diversity of Acridoidea in Different Grassland Types

Mountain meadow grasslands, temperate grasslands, and temperate desert grasslands contained more Acridoidea species than did other grassland types, with 42, 43, and 38 species, respectively. The number of Acridoidea specimens in these grassland types was also higher compared to other grassland types. The diversity indices of Acridoidea in temperate grasslands and mountain meadows were relatively high (3.053 and 2.563, respectively), with these two grassland types exhibiting both a higher number of Acridoidea species and a greater number of specimens. Beyond the availability of green vegetation, plant nutrients play a significant role in regulating grasshopper population dynamics [39]. This suggests that Acridoidea are more likely to thrive in grassland ecosystems characterized by abundant vegetation, ample food sources, and moderate temperature and humidity.

Different families, genera, and species of Acridoidea exhibit distinct preferences for specific grassland types and ecological environments. Acridoidea species of the family Arcypteridae are primarily concentrated in mountain meadows and temperate grasslands. This preference is attributed to their phytophilous nature [40], as these grassland types feature abundant plant species and suitable temperature and humidity conditions, creating an ideal environment for their survival and reproduction. In contrast, Acridoidea species of the family Oedipodidae are more commonly found in temperate deserts, temperate desert grasslands, and temperate steppe desert grasslands. These habitats, characterized by exposed rocky surfaces and sparse vegetation, provide optimal conditions for ground-dwelling and rock-dwelling Acridoidea of the Locustidae family to thrive [40].

The similarity of Acridoidea species across different grassland types reveals some notable differences in species composition, which may be attributed to factors such as anthropogenic grazing disturbances [41] and variations in the vertical distribution of plant species diversity. Additionally, the influence of hydrothermal resources on Acridoidea behaviors, including oviposition, hatching, molting, and reproduction, may contribute to variations in Acridoidea species composition, onset periods, and population characteristics [42]. Furthermore, abiotic environmental factors, such as food availability and quality, environmental coercion, and environmental changes, also play a significant role in shaping Acridoidea populations [43,44]. Acridoidea adapt to changes in the grassland microenvironment to enhance their capacity to cope with environmental fluctuations, leading to the development of distinct dominance mechanisms [45]. Overall, the heterogeneity of hydrothermal resources and vegetation characteristics across different grassland types significantly impacts the growth, habitat, feeding, and distribution patterns of Acridoidea. This, in turn, influences the spatial patterns of Acridoidea species composition, distribution, and diversity within various grassland ecosystems.

### 4.3. Species Diversity of Acridoidea at Different Altitudes

Soil factors play an important role in Orthoptera diversity [46]. Soil interventions have been shown to reduce locust damage [47]. This is consistent with the conclusion we reached: the dominant Acridoidea species are primarily found in areas with low-to-medium-low soil erodibility. These areas, which extend from parts of the Ili River Valley to parts of Tacheng, also exhibit higher grassland Acridoidea species diversity indices, particularly in the 9th, 5th, and 4th divisions. Several factors contribute to the higher Acridoidea species diversity index in these regions: favorable soil conditions, higher land use rates, increased vegetation cover, and stronger human intervention and planning. These factors promote the development of more stable ecosystems, enhance biodiversity, and provide a suitable ecological environment for the occurrence of grassland Acridoidea.

The vertical distribution of grassland Acridoidea shows that both the number of species and the species diversity index increase with altitudes in the range of 0–1500 m. However, as altitude rises from 1500 m to 3500 m, both the number of species and the species diversity index decrease, with the highest values observed between 1000 and 1500 m. This finding aligns with previous research, which concludes that biodiversity does not uniformly increase with altitude but peaks at intermediate elevations. Similar trends in biodiversity changes with altitude have been reported by Kessler [48,49,50]. Several factors may explain this pattern: First, grasslands at around 1500 m exhibit moderate temperatures and a variety of grassland types, including all meadow types except for alpine and lowland meadows. These areas also have expansive grasslands with healthy vegetation and relatively low levels of anthropogenic interference, providing a stable ecosystem for Acridoidea. Second, the greater variety of vegetation types at this altitude offers Acridoidea a favorable environment with abundant food resources, which support their growth, development, and reproduction [51].

The results of the present study are consistent with those reported by Qiao [52]. This study found that the Arcypteridae family is the dominant Acridoidea group at higher elevations. Similarly, Qiao concluded that Acridoidea from the genera *Chorthippus* sp., *Stenobothrus* sp., and *Omocestus* sp., all of which belong to the Arcypteridae family, are more commonly distributed in grasslands at higher elevations.

### 4.4. Effects of Temperature and Rainfall on Species Diversity of Acridoidea

According to the water–energy dynamic hypothesis [53], temperature and precipitation are important environmental factors for maintaining insect species richness [54]. Species in the order Orthoptera, as thermophiles, are extremely sensitive to climate change, and small fluctuations in temperature and precipitation can cause drastic changes in the distribution patterns of their taxa [55]. This study analyzed the correlations between Acridoidea species diversity and climatic factors and revealed significant positive correlations between species diversity and various climatic variables, including the average temperature of the wettest quarter (Bio-8), the average temperature of the warmest quarter (Bio-10), precipitation in the wettest month (Bio-13), and precipitation in the driest quarter (Bio-17). Higher ambient temperatures have been reported to favor the growth and development of Orthoptera, and they remain mostly categorized as driven by precipitation patterns [56], which aligns with the results of the present study [57].

These findings provide a stronger explanation for the conclusions drawn in the previous section, highlighting that the 4th, 5th, and 9th divisions exhibited higher levels of species diversity. The grasslands of the 4th division, located in the Ili River Valley, benefit from abundant perennial pastures and sufficient rainfall. The 5th and 9th divisions, situated to the north of the Tianshan Mountains, have relatively lower elevations and more favorable temperatures compared to the rest of Xinjiang. Warmer climates will likely allow grasshoppers to achieve body temperatures closer to optimal over a greater part of the day, allowing them to eat, digest, and grow faster [50]. The ecological conditions in these divisions support higher biodiversity, and the correlation analysis further substantiated the validity of these findings.

The spatial distribution pattern of species diversity has become a hot topic in recent research in ecology, biogeography, and conservation biology [58,59,60]. Future studies should aim to address gaps in the understanding of the relationship between grassland Acridoidea species diversity and habitats. Specifically, research should explore the effects of many factors, including abiotic factors (temperature, precipitation), food availability and quality, habitat availability and structure, symbionts, competitors, predators, pathogens, and interactions among these factors [61]. Furthermore, a more detailed clarification of the relationship between grassland Acridoidea species diversity and habitats is needed.

## 5. Conclusions

In this study, we first analyzed the species diversity of grassland Acridoidea across various divisions (regions) of the Xinjiang Production and Construction Corps, comparing the diversity indices of Acridoidea species in different divisions. Second, we conducted a comprehensive investigation into the effects of ecological factors on the species diversity and distribution of grassland Acridoidea, including the grassland type, altitude, temperature, and soil erodibility. Our findings revealed significant differences in Acridoidea community composition and diversity across different ecological environments within the Xinjiang Corps. These differences can be attributed to several factors, including anthropogenic grazing disturbances, variations in the vertical distribution of plant species, and the influence of water and heat resources on Acridoidea behaviors such as egg laying, hatching, and reproduction. Additionally, abiotic environmental factors, including environmental gradients, stresses, and changes, play a role in shaping the Acridoidea distribution. Ultimately, the distribution of Acridoidea in the grasslands managed by the Corps is regional, flexible, and diverse. In conclusion, the heterogeneity of regional ecological environments, shaped by the interplay of grassland type, altitude, soil characteristics, temperature, and humidity, significantly influences the growth, habitat, feeding, and distribution patterns of grassland Acridoidea, leading to a varied distribution of Acridoidea species across different habitats.

## Figures and Tables

**Table 1:** The grassland type and area of different regions and regiment fields.

Division Headquarter	Area	Grassland Type	Grassland Area	Regiment Field Distribution
1st division	Aksu prefecture	Temperate grasslands, alpine meadows, lowland meadows, and mountain meadows	2.3373	4th and 5th regiments
2nd division	Bayin’guoleng Mongol autonomous prefecture of Xinjiang	Temperate grasslands, alpine meadows, and lowland meadows	18.4	21st, 22nd, 24th, 25th, 27th, 29th, 30th, 36th, and 223rd regiments
3rd division	Kashgar or Kāshí prefecture	Alpine meadows, temperate desert grasslands, temperate meadow grasslands, temperate deserts, and lowland meadows	11.31	Toyun Ranch and Yecheng Second Ranch
4th division	Ili Kazakh autonomous prefecture	Temperate grasslands, temperate meadow grasslands, mountain meadows, lowland meadows, alpine meadows, temperate deserts, and temperate desert grasslands	19.17	61st, 63rd, 64th, 66th, 67th, 70th, 71st, 72nd, 73rd, 74th, 76th, 77th, 78th, and 79th regiments
5th division	Börtala Mongol autonomous prefecture	Temperate grasslands, temperate meadow grasslands, mountain meadows, lowland meadows, alpine meadows, temperate deserts, and temperate desert grasslands	13.16	83rd, 84th, 86th, 87th, 88th, 89th, 90th, and 91st regiments
6th division	Changji prefecture	Temperate grasslands, temperate deserts, temperate desert grasslands, and temperate steppe deserts	26.59	Beitashan Ranch, Hongqi Ranch, and Qitai Ranch
8th division		Temperate grasslands, temperate deserts, mountain meadows, temperate desert grasslands, lowland meadows, and alpine meadows	7.36	142nd, 143rd, and 151st regiments
12th division		Temperate grasslands, temperate meadow grasslands, mountain meadows, temperate desert grasslands, lowland meadows, alpine meadows, temperate deserts, and temperate steppe deserts	19.92	104th regiment
7th division	Tacheng prefecture	Temperate grasslands, temperate deserts, mountain meadows, lowland meadows, temperate meadow grasslands, temperate desert grasslands, and alpine meadows	10.44	124th, 131st, and 137th regiments
9th division		Lowland meadows, temperate grasslands, temperate deserts, temperate desert grasslands, mountain meadows, temperate meadow grasslands, alpine meadows, and temperate steppe deserts	13.65	161st, 162nd, 163rd, 164th, 165th, 166th, 167th, 168th, and 170th regiments
10th division	Altay prefecture	Temperate meadow grasslands, temperate grasslands, temperate deserts, temperate desert grasslands, mountain meadows, lowland meadows, alpine meadows, and temperate steppe deserts	9.29	181st, 182nd, 183rd, 184th, 185th, 186th, 187th, and 188th regiments
13th division	Kumul prefecture	Temperate grasslands, temperate desert grasslands, alpine meadows, temperate deserts, lowland meadows, and temperate steppe deserts	18.59	Red Hill Farm, Red Star Farm, Yellowfield Farm, and Willow Springs Farm
14th division	Khotan prefecture	Temperate grasslands, temperate desert grasslands, alpine meadows, temperate deserts, lowland meadows, and temperate steppe deserts	7.61	I Ranch, 47th regiment

**Table 2:** The families, genera, species, specimens, and dominant species (genera) of Acridoidea identified in each division.

Division	Number of Families	Number of Genera	Number of Species	Number of Specimens	Dominant Species
1st division	2	2	2	7	*Chorthippus biguttulus* *Gomphocerus sibiricus*
2nd division	2	4	7	68	*Myrmeleotettix palpalis*
3rd division	2	3	7	13	*Omocestus rufipes*
4th division	6	27	60	3130	*Dociostaurus brevicollis*
5th division	5	21	39	616	*Calliptamus barbarus* *Calliptamus italicus*
6th division	4	9	15	49	*Sphingonotus nebulosus* *Stauroderus scalaris*
7th division	4	12	22	224	*Gomphocerus sibiricus* *Omocestus haemorrhoidalis*
8th division	4	12	20	772	*Calliptamus italicus* *Omocestus haemorrhoidalis* *Notostaurus albicornis*
9th division	4	16	30	235	*Calliptamus barbarus* *Calliptamus italicus* *Oedaleus decorus*
10th division	3	7	11	55	*Bryodemella zaisanica ferrugineums* *Sphingonotus eurasius* *Myrmeleotettix brachypterus*
12th division	3	10	12	82	*Omocestus haemorrhoidalis* *Oedaleus decorus*
13th division	1	3	7	19	*Sphingonotus obscuratus*
14th division	1	3	6	20	*Chorthippus maritimus* *huabeiensis*

**Table 3:** The *Shannon-Wiener* diversity (H) and *Pielou*’s evenness (E) indices of locusts in grassland habitats of Xinjiang.

Division	H	E
1st division	0.598	0.863
2nd division	1.491	0.766
3rd division	1.692	0.87
4th division	2.883	0.704
5th division	2.979	0.813
6th division	2.169	0.800
7th division	2.349	0.759
8th division	2.167	0.730
9th division	2.795	0.822
10th division	2.17	0.905
12th division	2.025	0.815
13th division	1.837	0.944
14th division	1.657	0.925

**Table 4:** Distribution of eight Acridoidea families in different grassland types (number of species).

Grassland Types	Lowland Meadow	Alpine Meadow	Mountain Meadow	Temperate Grassland	Temperate Meadow Grassland	Temperate Desert	Temperate Desert Grassland	Temperate Steppe Desert
Arcypteridae	14	10	29	28	14	8	2	2
Oedipodidae	7	1	10	5	0	24	12	12
Gomphoceridae	3	3	1	5	0	0	0	0
Acrididae	0	1	1	2	0	1	0	0
Catantopidae	3	3	0	3	0	3	3	3
Pamphagidae	0	0	1	0	0	1	0	0
Chrotogonidae	0	0	0	0	0	1	0	0
Pyrgomorphidae	1	0	0	0	0	0	0	0
Number of genera	16	10	18	22	3	16	8	7
Number of species	28	18	42	43	14	38	17	17

**Table 5:** The number of species, specimens, and *Shannon–Wiener* (H) and *Pielou* (E) indices of Acridoidea in different grassland types.

Grassland Type	Number of Species	Number of Specimens	H	E
Lowland meadow	28	361	2.505	0.752
Alpine meadow	18	86	2.421	0.837
Mountain meadow	42	1257	2.563	0.686
Temperate grassland	43	1351	3.053	0.812
Temperate meadow grassland	14	53	2.055	0.778
Temperate deserts	38	1527	2.437	0.670
Temperate desert grassland	17	447	1.887	0.666
Temperate steppe desert	17	208	1.703	0.601

**Table 6:** The number of species, number of specimens, and *Shannon–Wiener* and *Pielou* indices of Acridoidea at different altitudes.

Altitude (Meters)	Number of Species	Number of Specimens	H	E
0–500	40	771	2.906	0.788
500–1000	52	1368	2.976	0.743
1000–1500	66	1831	3.237	0.725
1500–2000	35	917	2.867	0.806
2000–2500	26	185	2.634	0.806
2500–3000	20	132	2.568	0.815
3000–3500	18	86	2.421	0.837

## Data Availability

Data is contained within the article or Supplementary Materials.

## Supplementary Materials

The following supporting information can be downloaded at: https://www.mdpi.com/article/10.3390/biology14010014/s1, File S1: Composition of grasshoppers in grasslands of 1–14 divisions in Xinjiang (Number of Specimens); File S2: Grassland investigation sites information.
