# Supplementary material for: Influence of Grassland Habitats on Acridoidea (Orthoptera) Species Diversity in Different Divisions of the Xinjiang Production and Construction Corps
Source: Biology (Basel). 2024 Dec 27;14(1):14. doi: 10.3390/biology14010014 (PMC11762391; doi:10.3390/biology14010014)
Supplement: Supplementary file 1 [file biology-14-00014-s001.zip › File S1 Composition of grasshoppers in grasslands of 1-14 divisions in Xinjiangú¿Number of Specimensú⌐.pdf]

Table S1. Composition of grasshoppers in grasslands of 1-14 divisions in Xinjiang（Number of Specimens）

Families	Species	1st	2nd	3rd	4th	5th	6th	7th	8th	9th	10th	12th	13th	14th	Agg
Arcypteridae	*Arcyptera fusca* Pallas, 1773				1			3							4
	Chorthippus fallax Zubovski, 1900							1							1
	Chorthippus foveatus Hsia & Jin, 1982			1	2	1									4
	Chorthippus intermedius Bey-Bienko, 1926							4							4
	Chorthippus unicubitus Xia et Jin, 1982				1			3							4
	Chorthippus albomarginatus De Geer, 1773		4		248		1		7						260
	Chorthippus dichrous Eversmann				115	4		1	51		1				172
	Chorthippus karelini				2							2			4
	Chorthippus qingzangensis Yin, 1984				30	3			32						65
	Chorthippus apricarius L., 1758			1	93		5	11	6	1				5	122
	Chorthippus biguttulus L., 1758	2	12		49	5	1	16	10	4				2	101
	Chorthippus maritimus huabeiensis Xia et Jin, 1982		5	3	13			41						6	68
	Chorthippus mollis Charpentier, 1825			1	2									3	6
	Chorthippus longicornis Latreille, 1804				36	2				1					39
	Chorthippus rufucornus Zheng, 1988				1										1
	Dociostaurus brevicollis Eversmann, 1848				881	7	1			7					896
	Dociostaurus tartarus Uvarov, 1921				141	36			8	12					197
	Dociostaurus kraussi Ingeniky, 1897				33	8			1	15	3				60
	Eremippus comatus Mistshenko, 1951		4												4
	Eremippus miramae Tarbinsky, 1927								1	1					2
	Eremippus parvulus Mistshenko, 1951				1										1

	Eremippus simplex Eversmann, 1859			3				1					3	7
	Euchorthippus pulvinatus Fischer von Waldheim, 1846			85	1	1		3	3					93
	Kangacrisoides huochengensis Wang, Zheng & Niu, 2006			9			1							10
	Notostaurus albicornis Eversmann, 1848			211	42	1		183	4					441
	Notostaurus rubripes Eversmann, 1848			1	15						4			20
	Omocestus haemorrhoidalis Charpentier, 1825	2		223	17	2	28	79	5		12			368
	Omocestus petraeus Brisout-Barneville, 1855	6		1	3				1					11
	Omocestus rufipes Zetterstedt, 1821		5	32	6		5		3				1	52
	Omocestus viridulus Brisout-Barneville, 1855			58			5	2	2					67
	Stauroderus scalaris Fischer von Waldheim, 1846			204	7	15	6		2					234
	Stenobothrus carbonarius Eversmann			31	1									32
	Stenobothrus lineatus Panzer, 1796			13				48						61
	Stenobothrus newskii Zubovski, 1900									5				5
	Stenobothrus werneri Adelung, 1907			21					1					22
	Xinjiangacris rufitibis Zheng, 1993			8										8
Oedipodidae	*Acrotylus insubricus inficitus* Walker, 1870			3	15									18
	Aiolopus thalassinus Fabricius, 1781			7										7
	Bryodema gebleri Fischer de Waldheim, 1836				20		1				9			30
	Bryodemella nigripennis Zheng, Zhang &				14		1				4			19

Zeng											
Bryodemella tuberculata Fabricius					1						1
Bryodemella zaisanica			15						3		18
Bryodemella zaisanica ferrugineum			2				1	14		2	19
Celes variabilis Pallas, 1771							2				2
Dericorys roseipennis			1								1
Epacromius tergestinus Megerle von Mühlfeld		3									3
Mioscirtus wagneri Eversmann		24	4								28
Oedaleus decorus Germar, 1825		72	21		3	74	39		31		240
Oedipoda caerulescens Linnaeus, 1758		88	20		4	22	20		6	1	161
Oedipoda miniata Pallas, 1771		8	3				1				12
Pyrgodera armata Fischer de Waldheim, 1820			1				8				9
Sphingonotus carinatus Saussure, 1888		13					1				14
Sphingonotus coerulipes Uvarov, 1922	1	7		10			9			2	29
Sphingonotus coerulipes Uvarov, 1922										4	4
Sphingonotus elegans Mistshenko, 1936		13	36	3						2	54
Sphingonotus eurasius Mistshenko		17	6					6			29
Sphingonotus halophilus B．-Bienko, 1929		4	3				2	3			12
Sphingonotus maculatus Uvarov		5									5
Sphingonotus nebulosus Fischer-Waldheim, 1846		13	19	5			17	3	5		62
Sphingonotus obscuratus latissimus Uvarov			26					5		5	36
Sphingonotus octofasciatus Serville, 1839			8								8
Sphingonotus rubescens Walker, 1870										3	3

	Sphingonotus turcmenus B.-Bienko, 1951			1	13		1	6		4					25
	Sphingonotus tristrial				2										2
	Sphingonotus salinus Pallas, 1773					2									2
Catantopidae	*Calliptamus italicus* Linnaeus, 1758				133	104	1	3	183	28		3			455
	Calliptamus barbarus Costa, 1836				69	83	1		59	32		2			246
	Calliptamus coelesyriensis Giglio-Tos, 1893				32	2			1	4		1			40
	Conophyma oliva Huang, 2006				7										7
	Conophyma xinjiangensis Huang, 1982				1										1
	Conophyma zhaosuensis Huang, 1982				1										1
Gomphoceridae	*Egnatius apicalis* Stål, 1876									5					5
	Gomphoceroides xingjiangensis Zheng, Xi & Lian, 1992				24			18							42
	Gomphocerus sibiricus Linnaeus, 1767	5			4		1	62			8				80
	Myrmeleotettix brachypterus Liu, 1982										6				6
	Myrmeleotettix palpalis Zubovski, 1900		35		2	52									89
Acrididae	*Chrysochraon dispar* Germar, 1834				2										2
	Duroniella gracilis Uvarov, 1926				7										7
	Euthystira xinyuanensis Liu, 1981				6										6
Pamphagidae	*Asiotmethis zachajini* B.-Bienko										1				1
	Haplotropis brunneriana Saussure				1										1
Pyrgomorphidae	*Pyrgomorpha conica* Olivier, 1791								1						1
Chrotogonidae	*Chrotogonus turanicus* Kuthy, 1905					1									1
Agg		7	68	13	3130	616	49	224	772	235	55	82	19	20	5290
