# Supplementary material for: Influence of Grassland Habitats on Acridoidea (Orthoptera) Species Diversity in Different Divisions of the Xinjiang Production and Construction Corps
Source: Biology (Basel). 2024 Dec 27;14(1):14. doi: 10.3390/biology14010014 (PMC11762391; doi:10.3390/biology14010014)
Supplement: Supplementary file 1 [file biology-14-00014-s001.zip › File S2 Grassland investigation site information.pdf]

Table S2 Grassland investigation site information and completion time of the 1st division of Xinjiang Production and Construction Corps

	Longitude	Latitude	Altitude(m)	Completion time
1	80.0567	41.6519	2587.4501	2023-06-21
2	80.0645	41.6691	2699.6432	2023-06-21
3	80.0920	41.6922	2876.8098	2023-06-21
4	80.0864	41.7187	3081.2586	2023-06-21
5	80.5548	41.7226	2175.7000	2023-06-20
6	80.4922	41.6774	2105.9827	2022-06-20
7	80.4008	41.7445	2494.8399	2022-06-20
8	80.4128	41.7460	2476.7296	2022-06-20
9	80.4090	41.7588	2512.8770	2022-06-20
10	80.3970	41.7649	2621.1220	2024-06-20
11	80.0872	41.6073	2800.5380	2024-06-21
12	80.0866	41.7563	3148.350	2024-06-21

Table S3 Grassland investigation site information and completion time of the 2nd division of Xinjiang Production and Construction Corps

	Longitude	Latitude	Altitude(m)	Completion time
1	85.1332	42.8172	2906.8398	2024-07-10
2	85.1834	42.8541	2707.5400	2024-07-10
3	85.2081	42.8994	2664.4009	2024-07-10
4	85.2079	42.8995	2604.0500	2024-07-10
5	85.4922	42.8871	2582.4731	2024-07-10
6	85.2830	42.9659	2493.2200	2024-07-09
7	85.2823	42.9674	2561.6918	2024-07-09
8	85.2874	42.9955	2519.1600	2024-07-09
9	85.3032	43.0088	2610.6773	2024-07-09
10	85.3355	43.0095	2633.9686	2024-07-09
11	85.3731	43.0031	2584.6900	2024-07-09
12	85.3729	43.0309	2667.3702	2024-07-09
13	85.4199	43.0558	2650.4800	2024-07-09
14	85.4201	43.0558	2650.8800	2024-07-09
15	85.4943	43.0729	2812.5352	2024-07-09
16	85.5345	43.0435	2844.7863	2023-07-09
17	85.5485	43.0253	2736.8100	2023-07-09
18	85.4742	43.0210	2657.6400	2023-07-09
19	85.4740	43.0209	2734.1123	2023-07-09
20	85.4324	42.9984	2697.4649	2023-07-09
21	85.4335	42.9615	2666.1138	2023-07-09
22	85.3979	42.9415	2610.4504	2023-07-09

23	85.3472	42.9369	2516.9400	2023-07-09
24	85.3096	42.9555	2496.2200	2023-07-09
25	85.3096	42.9555	2496.0400	2023-07-09
26	89.5231	38.5691	3191.2800	2023-06-27
27	89.5467	38.5506	3209.5100	2023-06-27
28	89.5327	38.5219	3248.6100	2023-06-27
29	89.4234	38.5556	3172.2200	2023-06-27
30	89.4239	38.5561	3183.7100	2023-06-27
31	85.2825	42.9670	2487.0000	2022-08-07
32	85.2862	42.9947	2518.1800	2022-08-07
33	85.1817	42.8511	2760.7498	2022-08-06
34	85.2078	42.9013	2602.2100	2022-08-06
35	85.1281	42.8143	2910.9538	2022-08-05
36	85.3713	43.0266	2593.1200	2022-08-04
37	85.4162	43.0542	2677.1993	2022-08-04
38	85.3724	43.0015	2583.0500	2022-08-04
39	85.4941	43.0724	2764.0999	2022-08-04
40	85.3350	43.0095	2556.8300	2022-08-04
41	85.5345	43.0437	2797.4418	2022-08-04
42	85.3012	43.0099	2537.0500	2022-08-04
43	85.5484	43.0254	2761.4428	2022-08-04
44	85.4715	43.0210	2679.7155	2022-08-04
45	85.3090	42.9555	2485.9200	2022-08-04
46	85.4390	43.0050	2654.5868	2022-08-04

47	85.4110	42.9465	2556.6400	2022-08-04
48	85.4346	42.9632	2621.5764	2022-08-04
49	85.5491	42.7777	2763.1756	2022-08-03
50	85.5198	42.7659	2753.5921	2022-08-03
51	85.6393	42.8104	2583.9000	2022-08-03
52	85.6790	42.8069	2628.8700	2022-08-03
53	85.6975	42.8227	2706.4500	2022-08-03
54	85.5316	42.8222	2666.2281	2022-08-03
55	85.7567	42.8127	2956.9300	2022-08-03
56	85.7569	42.8125	2941.0800	2022-08-03
57	85.4366	42.8994	2555.3772	2022-08-03
58	85.4927	42.8730	2578.6292	2022-08-03
59	85.5406	42.8516	2607.3876	2022-08-03
60	85.4471	42.8454	2663.8300	2022-08-02
61	85.4688	42.8296	2693.9121	2022-08-02
62	85.5050	42.8386	2646.7209	2022-08-02
63	85.5221	42.8284	2677.7711	2022-08-02
64	85.5310	42.7971	2718.0092	2022-08-02
65	85.4891	42.7889	2817.5336	2022-08-02
66	85.4077	42.7819	2816.8360	2022-08-02
67	85.4386	42.8175	2727.9600	2022-08-02

Table S4 Grassland investigation site information and completion time of the 3rd division of Xinjiang Production and Construction Corps

	Longitude	Latitude	Altitude(m)	Completion time
1	77.3244	36.9890	3317.8866	2023-06-26
2	77.2882	37.0400	3089.5405	2023-06-26
3	77.2975	37.0681	2918.1600	2023-06-25
4	77.3055	37.1220	2717.7794	2023-06-25
5	77.2942	37.1332	2673.3120	2023-06-25
6	75.2839	40.3799	3542.5703	2023-06-23
7	75.3326	40.3908	3261.8111	2023-06-23
8	75.3491	40.3641	3150.7800	2023-06-23
9	75.3494	40.3624	3147.8300	2022-05-30
10	75.3216	40.3885	3299.2600	2022-05-30
11	75.2830	40.3808	3526.7200	2022-05-30
12	77.2989	37.1289	2656.3900	2022-05-26
13	77.3209	36.9927	3228.3600	2022-05-26
14	77.2894	37.0358	3052.6900	2022-05-26
15	77.2931	37.0641	2910.6300	2022-05-26
16	77.3077	37.1201	2708.4300	2022-05-26
17	77.3055	37.1220	2717.7794	2024-07-26
18	77.2942	37.1332	2673.3120	2024-07-26
19	75.2839	40.3799	3542.5703	2024-07-25
20	75.3326	40.3908	3261.8111	2024-07-25
21	75.3491	40.3641	3150.7800	2024-07-25

22	75.3494	40.3624	3147.8300	2024-07-23
23	75.3216	40.3885	3299.2600	2024-07-23
24	75.2830	40.3808	3526.7200	2024-07-23
25	77.2989	37.1289	2656.3900	2024-07-30
26	77.3209	36.9927	3228.3600	2024-07-30
27	77.2894	37.0358	3052.6900	2024-07-30
28	77.2931	37.0641	2910.6300	2024-07-26
29	77.3077	37.1201	2708.4300	2024-07-26
30	77.3244	36.9890	3317.8866	2024-07-26
31	77.2882	37.0400	3089.5405	2024-07-26
32	77.2975	37.0681	2918.1600	2024-07-26
33	77.3055	37.1220	2717.7794	2024-07-26
34	77.2942	37.1332	2673.3120	2024-07-26
35	75.2839	40.3799	3542.5703	2024-07-25

Table S5 Grassland investigation site information and completion time of the 4th division of Xinjiang Production and Construction Corps

	Longitude	Latitude	Altitude(m)	Completion time
1	80.6783	44.0362	586.1800	2023-07-29
2	80.7744	43.4061	1554.2900	2023-07-28
3	81.2531	44.1570	164.9700	2023-07-28
4	81.3595	44.3118	2024.2000	2023-07-27
5	81.3819	44.3150	2049.6000	2023-07-27
6	81.9103	43.5006	984.1800	2023-07-26
7	83.0903	43.6404	1690.5500	2023-07-25
8	83.2853	43.8214	1659.3000	2023-07-24
9	81.9500	42.9940	128.2100	2023-07-21
10	80.7855	43.0134	1767.9400	2023-07-20
11	80.6883	43.0662	1898.7100	2023-07-19
12	80.5890	42.9158	1662.6200	2023-07-19
13	81.7801	43.3746	2249.0500	2022-07-28
14	81.8064	43.3707	2212.4100	2022-07-28
15	81.8511	43.4897	1215.6900	2022-07-26
16	81.8975	43.5033	981.1600	2022-07-26
17	81.9178	43.4649	1101.3800	2022-07-26
18	81.8950	43.4469	1207.3800	2022-07-26
19	82.6834	43.4043	926.5900	2022-07-25
20	82.7217	43.4240	819.2000	2022-07-25
21	82.6053	43.1735	1302.8400	2022-07-25
22	82.6093	43.1728	1321.9500	2022-07-25

23	82.6280	43.1548	1285.8600	2022-07-25
24	82.6316	43.1527	1293.2000	2022-07-25
25	81.9512	42.9842	1791.9900	2022-07-22
26	81.9685	42.9541	1954.5100	2022-07-22
27	81.9713	42.9304	1974.2400	2022-07-22
28	81.9939	42.9588	1631.6300	2022-07-22
29	82.0370	42.9473	2083.3566	2022-07-22
30	82.0937	42.8938	2478.4000	2022-07-22
31	80.9143	43.0023	1650.2400	2022-07-21
32	80.8438	43.0305	1728.1700	2022-07-21
33	80.8108	43.0203	1740.1300	2022-07-21
34	80.8043	43.0117	1717.6700	2022-07-21
35	80.7777	43.0639	1810.5200	2022-07-21
36	80.8105	43.0713	1847.5102	2022-07-21
37	80.9921	43.0484	1770.5000	2022-07-21
38	80.8788	43.0764	1773.6600	2022-07-21
39	80.9368	43.0312	1676.7200	2022-07-21
40	80.8300	43.1068	1869.6500	2022-07-20
41	80.7579	43.1023	1940.9400	2022-07-20
42	80.8203	43.1353	2055.0000	2022-07-20
43	80.8435	43.1634	2109.0600	2022-07-20
44	80.8698	43.1287	1934.3100	2022-07-20
45	80.7439	43.0218	1862.5000	2022-07-19
46	80.7644	43.0302	1930.9413	2022-07-19

47	80.7641	43.0302	1879.0800	2022-07-19
48	80.7129	43.0212	1732.8000	2022-07-19
49	80.6858	43.0679	1883.8800	2022-07-19
50	80.6860	43.0682	1891.0400	2022-07-19
51	80.5898	43.0169	1863.9000	2022-07-19
52	80.5942	42.9854	1721.6600	2022-07-19
53	80.5457	42.9635	1743.5900	2022-07-19
54	80.6851	42.9215	1630.0200	2022-07-18
55	80.6416	42.9035	1636.6000	2022-07-18
56	80.5786	42.9236	1658.9400	2022-07-18
57	80.5333	42.9405	1718.2900	2022-07-18
58	80.4788	42.9545	1851.6300	2022-07-18
59	80.4197	42.9916	1962.1500	2022-07-18
60	80.4069	43.0225	2154.3600	2022-07-18
61	80.4795	43.0261	2295.8400	2022-07-18
62	80.5446	43.0377	2034.4700	2022-07-18
63	80.6000	43.0552	1966.9600	2022-07-18
64	80.6055	43.0572	1965.6100	2022-07-18
65	80.6055	43.0572	1969.0500	2022-07-18
66	80.6632	43.0824	1986.8900	2022-07-18
67	80.7202	43.0908	1946.6000	2022-07-18
68	80.2926	42.7733	1679.6900	2022-07-16
69	80.2722	42.7896	1695.7500	2022-07-16
70	80.3088	42.8185	1719.8573	2022-07-16

71	80.3783	42.8267	1655.2200	2022-07-16
72	80.4564	42.8542	1657.5100	2022-07-16
73	80.5641	42.7446	1661.7700	2022-07-16
74	80.4330	42.8205	1662.5800	2022-07-16
75	81.5882	44.1707	1880.0900	2022-07-14
76	81.5883	44.1702	1888.0500	2022-07-14
77	81.6002	44.2091	1895.0100	2022-07-14
78	80.7581	43.4895	1365.8700	2022-07-08
79	80.7727	43.4504	1343.4400	2022-07-08
80	80.7986	43.3959	1532.1400	2022-07-08
81	80.8560	43.3792	1691.2500	2022-07-08
82	80.7551	43.3792	1792.8200	2022-07-08
83	80.7823	43.4114	1486.4300	2022-07-08
84	80.7823	43.4114	1483.8100	2022-07-08
85	80.6022	43.8269	478.7600	2022-07-07
86	80.6134	43.8142	490.0400	2022-07-07
87	80.6135	43.8141	488.8800	2022-07-07
88	80.5976	43.8001	491.1100	2022-07-07
89	80.5864	43.8093	524.5222	2022-07-07
90	83.7080	43.6754	1875.5000	2022-06-27
91	83.7084	43.6764	1901.8700	2022-06-27
92	83.7084	43.6760	1896.4700	2022-06-27
93	83.7084	43.6760	1897.0100	2022-06-27
94	83.7082	43.6761	1934.6100	2022-06-27

95	83.7082	43.6761	1934.6100	2022-06-27
96	83.6479	43.6967	1718.6800	2022-06-27
97	83.6480	43.6967	1720.3000	2022-06-27
98	83.6388	43.7078	1774.7300	2022-06-27
99	83.6609	43.6838	1786.9200	2022-06-27
100	83.6255	43.7080	1774.7300	2022-06-27
101	83.6255	43.7080	1764.9900	2022-06-27
102	83.0024	43.7676	1498.4300	2022-06-26
103	83.0415	43.7286	1456.2200	2022-06-26
104	83.0572	43.6849	1559.4200	2022-06-26
105	83.0572	43.6849	1560.3400	2022-06-26
106	83.0572	43.6849	1560.2900	2022-06-26
107	83.0572	43.6848	1562.0100	2022-06-26
108	83.0879	43.6446	1686.3400	2022-06-26
109	83.1091	43.6451	1751.5700	2022-06-26
110	83.1089	43.6450	1744.3000	2022-06-26
111	83.1085	43.6456	1771.3400	2022-06-26
112	83.1086	43.6459	1762.2800	2022-06-26
113	83.1079	43.6459	1796.6800	2022-06-26
114	83.2602	43.7807	1512.7700	2022-06-25
115	83.2602	43.7807	1512.7700	2022-06-25
116	83.2602	43.7806	1512.9800	2022-06-25
117	83.2864	43.8087	1600.6800	2022-06-25
118	83.2864	43.8088	1602.7100	2022-06-25

119	83.2864	43.8088	1601.5600	2022-06-25
120	83.2864	43.8087	1602.0800	2022-06-25
121	83.2827	43.8185	1615.5800	2022-06-25
122	83.2829	43.8187	1618.5100	2022-06-25
123	83.3324	43.8748	1779.0700	2022-06-25
124	83.3305	43.8765	1833.4900	2022-06-25
125	83.3319	43.8744	1769.1600	2022-06-25
126	83.3117	43.8542	1701.3100	2022-06-25
127	84.3912	43.4324	2927.2000	2022-06-24
128	84.4399	43.4537	3135.0200	2022-06-24
129	84.0803	43.2282	1986.9600	2022-06-24
130	83.9386	43.2082	0.0000	2022-06-24
131	84.1030	43.2158	2049.5300	2022-06-24
132	84.1034	43.2160	2064.5300	2022-06-24
133	84.1035	43.2161	2052.3300	2022-06-24
134	84.1237	43.2012	2156.7100	2022-06-24
135	83.4106	43.5624	1241.1600	2022-06-23
136	83.4106	43.5625	1237.9800	2022-06-23
137	83.4121	43.5626	1305.0000	2022-06-23
138	83.4112	43.5626	1254.6300	2022-06-23
139	83.4111	43.5625	1244.2900	2022-06-23
140	83.4309	43.5635	1710.7100	2022-06-23
141	83.4443	43.5784	1939.9400	2022-06-23
142	83.4442	43.5782	1940.5000	2022-06-23

143	83.4442	43.5781	1944.3900	2022-06-23
144	80.8687	44.5718	2216.1300	2022-06-22
145	80.8563	44.5781	2230.8700	2022-06-22
146	81.4022	44.3056	2038.2600	2022-06-21
147	81.3828	44.3148	2062.0500	2022-06-21
148	81.3830	44.3152	2053.3800	2022-06-21
149	81.3582	44.3119	2026.5700	2022-06-21
150	81.3583	44.3119	2022.3500	2022-06-21
151	81.3582	44.3119	2022.1500	2022-06-21
152	81.3583	44.3119	2017.6700	2022-06-21
153	81.0949	44.0378	947.9600	2022-06-20
154	81.0949	44.0378	947.9600	2022-06-20
155	81.2659	44.1310	878.1000	2022-06-20
156	81.2807	44.2297	1381.5500	2022-06-20
157	81.2974	44.2289	1537.4800	2022-06-20
158	81.2974	44.2290	1533.1600	2022-06-20
159	81.2674	44.2089	1202.1600	2022-06-20
160	81.2674	44.2084	1212.7500	2022-06-20
161	81.2676	44.2081	1215.2000	2022-06-20
162	81.2677	44.2079	1209.7200	2022-06-20
163	81.2675	44.1951	1221.4900	2022-06-20
164	81.2667	44.1952	1204.0800	2022-06-20
165	81.2669	44.1956	1206.9000	2022-06-20
166	81.2671	44.1956	1215.3000	2022-06-20

167	81.2676	44.1960	1215.8900	2022-06-20
168	81.2677	44.1955	1214.4100	2022-06-20
169	81.2492	44.1555	1128.3500	2022-06-20
170	81.2471	44.1508	1107.2900	2022-06-20
171	80.6909	44.0460	559.7900	2022-06-19
172	80.6891	44.0324	562.5400	2022-06-19
173	80.6696	44.0381	562.5600	2022-06-19
174	80.6699	44.0383	562.0400	2022-06-19
175	80.4818	44.3156	1115.8400	2022-06-18
176	80.4464	44.3769	1381.0400	2022-06-18
177	80.4459	44.3768	1380.0000	2022-06-18
178	80.4406	44.2877	979.4500	2022-06-18
179	80.4214	44.3534	1278.4000	2022-06-18
180	80.4212	44.3535	1276.5100	2022-06-18
181	80.4442	44.3828	1395.1500	2022-06-18
182	80.4282	44.4143	1682.2600	2022-06-18
183	80.4282	44.4143	1683.7700	2022-06-18
184	80.4275	44.4136	1658.4500	2022-06-18
185	80.4225	44.4631	2189.1200	2022-06-18
186	83.0024	43.7676	1498.4300	2024-06-20
187	83.0415	43.7286	1456.2200	2024-06-20
188	83.0572	43.6849	1559.4200	2024-06-20
189	83.0572	43.6849	1560.3400	2024-06-20
190	83.0572	43.6849	1560.2900	2024-06-19

191	83.0572	43.6848	1562.0100	2024-06-19
192	83.0879	43.6446	1686.3400	2024-06-19
193	83.1091	43.6451	1751.5700	2024-06-19
194	83.1089	43.6450	1744.3000	2024-06-18
195	83.1085	43.6456	1771.3400	2024-06-18
196	83.1086	43.6459	1762.2800	2024-06-18
197	83.1079	43.6459	1796.6800	2024-06-18
198	83.2602	43.7807	1512.7700	2024-06-18
199	80.8687	44.5718	2216.1300	2024-06-18
200	80.8563	44.5781	2230.8700	2024-06-18
201	81.4022	44.3056	2038.2600	2024-06-18
202	81.3828	44.3148	2062.0500	2024-06-18
203	81.3830	44.3152	2053.3800	2024-06-18
204	81.3582	44.3119	2026.5700	2024-06-18
205	81.3583	44.3119	2022.3500	2024-06-20
206	81.3582	44.3119	2022.1500	2024-06-20
207	81.3583	44.3119	2017.6700	2024-06-20
208	81.0949	44.0378	947.9600	2024-06-20
209	81.0949	44.0378	947.9600	2024-06-19

Table S6 Grassland investigation site information and completion time of the 5th division of Xinjiang Production and Construction Corps

	Longitude	Latitude	Altitude(m)	Completion time
1	81.2946	44.7371	2008.7600	2024-06-10
2	81.3398	44.7606	1759.7900	2024-06-10
3	81.3668	44.8114	1532.9900	2024-06-10
4	81.4345	45.1856	1606.5300	2024-06-10
5	81.4345	45.1856	1606.5300	2024-06-10
6	81.4534	45.1697	1490.4800	2024-06-10
7	81.4437	45.1976	1679.5100	2024-06-10
8	81.5134	45.1921	1587.7500	2024-06-10
9	81.5052	45.1572	1331.5900	2024-06-10
10	79.9848	44.8639	2978.9000	2024-06-10
11	79.9848	44.8639	2978.9000	2024-06-10
12	79.9881	44.8652	2935.9000	2023-06-14
13	80.0350	44.8849	2732.6500	2023-06-14
14	80.0985	44.9163	2530.2100	2023-06-14
15	80.2012	44.9385	2338.9200	2023-06-14
16	80.3040	44.9466	2212.4500	2023-06-14
17	80.3679	44.9538	2122.5300	2023-06-14
18	80.4418	44.9492	2028.4100	2023-06-14
19	80.5479	44.9576	1891.9400	2023-06-14
20	80.5793	44.9612	1852.2100	2023-06-14
21	80.6674	44.9805	1735.9000	2023-06-14
22	80.7596	45.0012	1634.4600	2023-06-14

23	80.9549	44.6349	2106.6800	2023-06-13
24	80.9177	44.6127	2148.3400	2023-06-13
25	80.8875	44.6044	2201.2700	2023-06-13
26	80.8009	44.6094	2432.3400	2023-06-13
27	80.8396	44.5771	2286.6600	2023-06-13
28	80.8782	44.5756	2193.0100	2023-06-13
29	82.2007	45.1557	984.3000	2023-06-12
30	82.2799	45.1976	1226.9700	2023-06-12
31	82.4737	45.1237	422.1300	2023-06-12
32	82.4403	45.1579	548.0100	2023-06-12
33	82.3485	45.1741	953.2900	2023-06-12
34	82.3941	45.1152	871.5100	2023-06-12
35	82.3717	45.0756	798.9000	2023-06-12
36	82.3860	45.0546	732.8600	2023-06-12
37	82.3775	45.0253	638.7800	2023-06-12
38	82.3775	45.0253	638.7800	2023-06-12
39	82.3312	45.0310	618.7100	2023-06-12
40	82.2649	45.0292	569.5300	2023-06-12
41	82.2651	45.0292	570.8200	2023-06-12
42	82.2424	45.1021	755.5000	2023-06-12
43	83.3586	44.3814	2680.6800	2023-06-11
44	83.3497	44.3777	2902.9627	2022-07-08
45	83.3686	44.3937	2601.9454	2022-07-08
46	82.1991	45.1634	1018.8000	2022-07-07

47	82.2727	45.1955	1220.5000	2022-07-07
48	81.2932	44.7684	1973.4417	2022-07-06
49	81.4332	45.1817	1567.9000	2022-07-01
50	80.7652	45.0026	1634.6900	2022-06-30
51	80.7652	45.0026	1634.7100	2022-06-30
52	80.6579	44.9786	1748.5900	2022-06-30
53	80.5765	44.9607	1852.9600	2022-06-30
54	80.5506	44.9581	1883.2800	2022-06-30
55	80.8733	45.0012	1489.0300	2022-06-30
56	80.5405	44.9574	1896.4500	2022-06-30
57	80.4411	44.9491	2024.6200	2022-06-30
58	80.3714	44.9537	2110.1000	2022-06-30
59	80.3060	44.9470	2204.1500	2022-06-30
60	80.1969	44.9379	2455.7613	2022-06-30
61	80.0991	44.9166	2524.5900	2022-06-30
62	80.0269	44.8807	2763.3100	2022-06-30
63	79.9845	44.8636	2986.6000	2022-06-30
64	80.9548	44.6348	2191.3753	2022-06-29
65	80.9257	44.6148	2132.2200	2022-06-29

Table S7 Grassland investigation site information and completion time of the 6th division of Xinjiang Production and Construction Corps

	Longitude	Latitude	Altitude(m)	Completion time
1	88.7319	43.8858	1572.3400	2023-07-23
2	89.0442	43.8889	1102.6000	2023-07-22
3	89.0442	43.8889	1102.7600	2023-07-22
4	89.3777	44.1138	606.9000	2023-07-22
5	89.3277	44.1215	593.2300	2023-07-22
6	88.9602	44.1750	540.5000	2023-07-22
7	89.0432	44.1672	544.5300	2023-07-22
8	89.0433	44.1672	544.5700	2023-07-22
9	89.0630	44.1702	544.9700	2023-07-22
10	89.0939	44.1524	552.1700	2023-07-22
11	89.1024	44.1725	544.6800	2023-07-22
12	89.0993	44.1856	537.6500	2023-07-22
13	90.6361	45.4925	1316.9000	2022-07-21
14	90.6159	45.5184	1289.6000	2022-07-21
15	90.5962	45.5430	1220.9000	2022-07-21
16	90.5672	45.5701	1118.7000	2022-07-21
17	90.8052	45.1556	2208.1900	2022-07-20
18	90.7830	45.1206	1716.9000	2022-07-20
19	90.7571	45.0730	1440.7000	2022-07-20
20	90.7340	45.0367	1216.8000	2022-07-20
21	90.6773	45.0313	1094.3000	2022-07-20
22	90.6345	45.0569	1125.1000	2022-07-20

23	90.6102	45.1046	1266.0900	2022-07-20
24	90.5638	45.1625	1380.4000	2022-07-20
25	90.5147	45.2316	1466.9000	2022-07-20
26	90.8868	45.1717	3127.1000	2022-07-20
27	91.0064	45.1355	2894.5600	2022-07-20
28	91.0899	45.1615	2654.9900	2022-07-20
29	90.7121	45.3942	1647.5000	2022-07-19
30	90.7742	45.3752	1712.8000	2022-07-19
31	90.7905	45.3523	1697.5000	2022-07-19
32	90.8003	45.3219	1909.7000	2022-07-19
33	90.8111	45.2685	2223.1000	2022-07-19
34	90.8590	45.2063	3201.9000	2022-07-19
35	90.7976	45.2181	2659.0000	2022-07-19
36	90.7081	45.2584	2523.7900	2022-07-19
37	90.5688	45.4366	1544.5675	2022-07-18
38	90.5688	45.4366	1582.6492	2022-07-18
39	90.5740	45.4860	1395.9017	2022-07-18
40	90.5740	45.4860	1432.7246	2022-07-18
41	90.6605	45.4627	1419.6000	2022-07-18
42	90.6757	45.4187	1553.6000	2022-07-18
43	90.6306	45.4402	1455.4000	2022-07-18
44	90.6309	45.4401	1613.3396	2022-07-18
45	90.6020	45.4130	1683.4902	2022-07-18
46	90.5771	45.3758	1743.4728	2022-07-18

47	90.6018	45.3184	1718.0000	2022-07-18
48	90.5626	45.3363	1717.9178	2022-07-18
49	90.5844	45.2858	1815.8542	2022-07-18
50	90.6380	45.2875	2079.4139	2022-07-18
51	90.5458	45.3115	1608.6000	2022-07-17
52	90.4613	45.3259	1444.6000	2022-07-17
53	90.4184	45.3245	1415.7000	2022-07-17
54	90.4824	45.3993	1379.4000	2022-07-17
55	90.5211	45.4659	1272.0000	2022-07-17
56	90.5323	45.5499	1140.4000	2022-07-17
57	90.5387	45.5072	1197.5000	2022-07-17
58	90.3850	45.5216	1185.5000	2022-07-17
59	90.4672	45.5233	1196.6000	2022-07-17
60	89.2170	43.9192	824.3000	2022-07-16
61	89.2129	43.9002	1122.1693	2022-07-16
62	89.1966	43.8870	985.8000	2022-07-16
63	89.0574	43.9098	1024.7000	2022-07-16
64	89.0262	43.9055	1076.2000	2022-07-16
65	89.0653	43.9308	985.5000	2022-07-16
66	89.0445	43.8879	1114.8000	2022-07-16
67	88.8867	44.0447	752.7000	2022-07-16
68	89.3279	44.1214	592.2900	2022-07-14
69	89.3405	44.0879	612.0900	2022-07-14
70	89.3824	44.0784	625.4000	2022-07-14

71	89.3966	44.1163	600.2900	2022-07-14
72	89.0669	44.2061	521.7000	2022-07-14
73	89.1020	44.1756	528.8000	2022-07-14
74	89.1121	44.1431	553.1000	2022-07-14
75	89.0442	44.1633	548.9000	2022-07-14
76	88.9876	44.1945	529.7000	2022-07-14
77	88.9699	44.1769	541.6000	2022-07-14

Table S8 Grassland investigation site information and completion time of the 7th division of Xinjiang Production and Construction Corps

	Longitude	Latitude	Altitude(m)	Completion time
1	84.8248	44.0666	1956.7500	2022-08-04
2	84.8558	44.0310	2326.6700	2022-08-04
3	84.8527	44.0291	2361.9600	2022-08-04
4	84.0362	44.1791	2321.0000	2022-08-03
5	83.6579	44.2358	2436.6500	2022-08-02
6	85.1231	46.8235	1521.2900	2022-07-25
7	85.0719	46.8258	1607.8500	2022-07-25
8	85.0441	46.8203	1591.2900	2022-07-25
9	85.0431	46.8147	1579.8000	2022-07-25
10	85.0102	46.8218	1647.6500	2022-07-25
11	85.0100	46.8238	1656.5700	2022-07-25
12	84.9922	46.8419	1826.8400	2022-07-25
13	84.9736	46.8448	1894.2600	2022-07-25
14	84.9695	46.8437	1886.1400	2022-07-25
15	85.0976	46.8730	1727.1100	2022-07-24
16	84.8952	46.8824	1897.1000	2022-07-24
17	84.8499	46.9240	1691.9900	2022-07-24
18	84.8742	46.8156	2122.6900	2022-07-24
19	84.8828	46.8266	2109.5900	2022-07-24
20	84.8434	46.8252	2086.9000	2022-07-24
21	85.1874	46.9055	1662.4500	2022-07-23
22	85.1893	46.9071	1663.1200	2022-07-23
23	85.2255	46.9887	2058.7900	2022-07-23

Table S9 Grassland investigation site information and completion time of the 8th division of Xinjiang Production and Construction Corps

	Longitude	Latitude	Altitude(m)	Completion time
1	85.3151	43.9500	1571.5100	2023-06-27
2	85.3199	43.9453	1636.9000	2023-06-27
3	85.3499	43.9296	1569.6000	2023-06-27
4	85.8580	43.9857	1266.9300	2023-06-26
5	85.8652	44.0273	897.8800	2023-06-26
6	85.8976	44.0209	950.5300	2023-06-26
7	85.6332	43.8722	1950.3900	2022-07-05
8	85.6332	43.8722	1950.9700	2022-07-05
9	85.6331	43.8722	1952.7600	2022-07-05
10	85.7975	43.9730	1075.3200	2022-06-15
11	85.8624	43.9713	1236.0300	2022-06-15
12	85.8767	43.9684	1137.4000	2022-06-15
13	85.9930	44.0305	858.8400	2022-06-15
14	85.9931	44.0305	863.5800	2022-06-15
15	86.0305	44.0780	787.1300	2022-06-15
16	86.0697	44.1221	705.4500	2022-06-15
17	86.1014	44.1579	616.5300	2022-06-15
18	85.8403	44.0271	912.7900	2022-06-14
19	85.8404	44.0279	912.9200	2022-06-14
20	85.7946	44.0268	885.2100	2022-06-14
21	85.7465	43.9897	975.6700	2022-06-14
22	85.7690	43.9685	1023.9700	2022-06-14

Table S10 Grassland investigation site information and completion time of the 9th division of Xinjiang Production and Construction Corps

	Longitude	Latitude	Altitude(m)	Completion time
1	84.5065	46.3957	1627.1400	2023-07-11
2	83.8178	46.8686	1117.9500	2023-07-09
3	83.8909	46.8526	90.3400	2023-07-09
4	82.4100	45.7431	981.4000	2023-07-08
5	82.5968	46.1177	913.1100	2023-07-08
6	82.6983	46.1223	1069.2800	2023-07-08
7	82.8049	46.5245	377.5900	2023-07-07
8	82.8663	46.7869	568.5900	2023-07-07
9	82.9967	47.0343	131.5000	2023-07-07
10	82.9719	47.0056	972.7200	2023-07-07
11	83.8064	45.6535	1705.3000	2022-08-01
12	83.7877	45.6484	1697.5000	2022-08-01
13	83.7713	45.6490	1733.0000	2022-08-01
14	83.7710	45.6566	1730.2000	2022-08-01
15	83.7453	45.6501	1736.8000	2022-08-01
16	83.7453	45.6500	1695.1100	2022-08-01
17	83.7422	45.6682	1732.4100	2022-08-01
18	83.7418	45.6719	1741.0200	2022-08-01
19	83.7499	45.6838	1803.0000	2022-08-01
20	83.7492	45.6944	1746.1700	2022-08-01
21	83.9179	45.5111	1370.3400	2022-08-01
22	83.7514	45.7038	1775.8000	2022-08-01

23	83.7617	45.7026	1798.1000	2022-08-01
24	83.7872	45.6984	1724.3000	2022-08-01
25	83.9150	45.5302	1392.9800	2022-08-01
26	83.8027	45.7012	1700.5000	2022-08-01
27	83.8994	45.5396	1436.0200	2022-08-01
28	83.8229	45.7026	1673.9000	2022-08-01
29	83.8873	45.5376	1449.9000	2022-08-01
30	83.8268	45.6895	1687.5000	2022-08-01
31	83.8317	45.6810	1687.1000	2022-08-01
32	83.8294	45.6720	1682.1000	2022-08-01
33	83.8360	45.6501	1630.9000	2022-08-01
34	83.8392	45.6317	1646.9000	2022-08-01
35	83.8422	45.6139	1623.9000	2022-08-01
36	83.8764	45.5385	1469.8800	2022-08-01
37	83.8554	45.5414	1487.1300	2022-08-01
38	83.8581	45.5253	1468.8500	2022-08-01
39	83.8893	45.5032	1403.8000	2022-08-01
40	83.8608	45.5070	1436.2400	2022-08-01
41	83.8473	45.8795	1692.0000	2022-07-31
42	83.8470	45.8802	1682.7000	2022-07-31
43	83.8646	45.8594	1677.5000	2022-07-31
44	83.8646	45.8594	1688.5000	2022-07-31
45	83.8943	45.8925	1626.3000	2022-07-31
46	83.8729	45.9081	1693.7000	2022-07-31

47	83.8582	45.9192	1659.6000	2022-07-31
48	84.5091	46.4260	2168.6900	2022-07-30
49	84.4920	46.4375	2040.3500	2022-07-30
50	84.4817	46.4397	2093.0600	2022-07-30
51	84.5366	46.4292	2150.5000	2022-07-30
52	84.5363	46.4293	2130.6000	2022-07-30
53	84.5443	46.4379	2044.3000	2022-07-30
54	84.5501	46.4479	1957.2000	2022-07-30
55	84.4645	46.4812	1598.9700	2022-07-30
56	84.5775	46.4536	1891.5000	2022-07-30
57	84.4580	46.4679	1725.6500	2022-07-30
58	84.4601	46.4630	1660.7400	2022-07-30
59	84.4586	46.4491	1804.5200	2022-07-30
60	84.5994	46.4467	1939.3000	2022-07-30
61	84.6171	46.4404	2029.8000	2022-07-30
62	84.4627	46.4269	2050.3500	2022-07-30
63	84.6350	46.4383	2068.6900	2022-07-30
64	84.6433	46.4365	2104.6900	2022-07-30
65	84.4578	46.4238	2007.9000	2022-07-30
66	84.6595	46.4368	1937.7000	2022-07-30
67	84.6776	46.4345	1808.9000	2022-07-30
68	84.6984	46.4318	1589.2000	2022-07-30
69	84.6996	46.4323	1623.8000	2022-07-30
70	84.4554	46.4061	1887.2800	2022-07-30

71	84.4529	46.4116	1948.7700	2022-07-30
72	84.7165	46.4304	1460.9000	2022-07-30
73	84.7271	46.4268	1358.7000	2022-07-30
74	84.7241	46.4117	1247.2000	2022-07-30
75	84.7413	46.4089	1161.5900	2022-07-30
76	84.4887	46.4077	1740.7400	2022-07-30
77	84.5061	46.3959	1622.8900	2022-07-30
78	84.5200	46.3904	1674.4200	2022-07-30
79	84.5345	46.3839	1479.8100	2022-07-30
80	84.7549	46.4015	1076.7000	2022-07-30
81	84.5423	46.3815	1414.5800	2022-07-30
82	84.5547	46.3749	1307.7200	2022-07-30
83	84.5544	46.3639	1155.9700	2022-07-30
84	82.4008	45.7736	884.3000	2022-07-28
85	82.4025	45.7756	892.9400	2022-07-28
86	82.4134	45.7596	1054.8000	2022-07-28
87	82.4089	45.7425	1025.9000	2022-07-28
88	82.3867	45.7455	947.3000	2022-07-28
89	82.3868	45.7454	903.3800	2022-07-28
90	82.3836	45.7343	946.9000	2022-07-28
91	82.3815	45.7184	965.9000	2022-07-28
92	82.3882	45.7067	970.3000	2022-07-28
93	82.3748	45.6641	944.8000	2022-07-28
94	82.3722	45.6569	933.5000	2022-07-28

95	82.3821	45.6488	938.0000	2022-07-28
96	82.3997	45.6373	992.8000	2022-07-28
97	82.4468	45.6363	1082.9000	2022-07-28
98	82.4709	45.6428	1156.7000	2022-07-28
99	82.5105	45.6646	1456.6000	2022-07-28
100	82.4667	45.6639	1256.7000	2022-07-28
101	82.4433	45.6645	1163.4000	2022-07-28
102	82.4343	45.6677	1065.7900	2022-07-28
103	82.4109	45.6779	1045.4000	2022-07-28
104	82.3942	45.6816	1007.5000	2022-07-28
105	82.3811	45.6973	962.5000	2022-07-28
106	82.3868	45.7070	982.8000	2022-07-28
107	82.3869	45.7070	982.6000	2022-07-28
108	82.3868	45.7069	985.4000	2022-07-28
109	82.3775	45.7151	946.4000	2022-07-28
110	82.3658	45.7141	878.3000	2022-07-28
111	82.3462	45.7159	857.3000	2022-07-28
112	82.3462	45.7159	857.3000	2022-07-28
113	82.8062	46.1205	0.0000	2022-07-28
114	82.5915	46.0430	1047.0900	2022-07-27
115	82.5635	46.0637	974.2000	2022-07-27
116	82.5707	46.0801	947.5000	2022-07-27
117	82.5689	46.0813	938.6000	2022-07-27
118	82.5688	46.0813	944.2000	2022-07-27

119	82.5829	46.0849	840.6000	2022-07-27
120	82.5833	46.0852	836.0000	2022-07-27
121	82.5761	46.1011	767.2000	2022-07-27
122	82.6052	46.1385	942.6000	2022-07-27
123	82.6052	46.1385	942.6000	2022-07-27
124	82.6954	46.1279	1060.0900	2022-07-27
125	82.7350	46.1163	1085.2000	2022-07-27
126	82.6784	46.0041	1262.0900	2022-07-27
127	82.6626	45.9752	1273.7000	2022-07-27
128	82.5949	45.9511	1094.2000	2022-07-27
129	82.5931	45.9508	1089.4000	2022-07-27
130	82.5504	45.9568	932.7000	2022-07-27
131	82.5067	45.9586	892.6000	2022-07-27
132	82.4987	45.9378	943.7000	2022-07-27
133	82.4268	45.9287	884.4000	2022-07-27
134	82.4268	45.9287	884.4000	2022-07-27
135	82.4277	45.9107	791.6000	2022-07-27
136	82.3990	45.9082	777.0000	2022-07-27
137	82.3871	45.9084	797.0000	2022-07-27
138	82.3748	45.8884	742.0000	2022-07-27
139	82.3664	45.8717	733.7000	2022-07-27
140	82.3665	45.8717	727.8000	2022-07-27
141	82.3817	45.8225	823.7000	2022-07-27
142	82.3825	45.7907	810.1900	2022-07-27

143	82.3732	45.7709	779.7100	2022-07-27
144	82.3449	45.7466	785.8500	2022-07-27
145	82.3428	45.7276	774.1700	2022-07-27
146	82.3299	45.7084	756.7000	2022-07-27
147	82.7894	46.5195	403.6000	2022-07-26
148	82.7814	46.5175	400.6000	2022-07-26
149	82.8548	46.7841	546.3600	2022-07-26
150	82.8099	46.7206	488.3000	2022-07-26
151	83.6999	46.8632	1192.0900	2022-07-25
152	83.6991	46.8631	1195.0900	2022-07-25
153	83.6987	46.8631	1196.7000	2022-07-25
154	83.6987	46.8631	1196.7000	2022-07-25
155	83.6761	46.9014	1460.0000	2022-07-25
156	83.6737	46.8479	1096.5900	2022-07-25
157	83.8840	46.8599	1036.9000	2022-07-24
158	83.8481	46.9049	1368.8000	2022-07-24
159	83.9127	46.8777	1111.2000	2022-07-24
160	83.9082	46.8808	1093.5900	2022-07-24
161	83.8738	46.9069	1304.0900	2022-07-24
162	83.8706	46.9345	1558.1000	2022-07-24
163	83.8036	46.9571	2103.5000	2022-07-24
164	83.8302	46.9467	2010.7000	2022-07-24
165	84.1241	46.8682	907.6000	2022-07-23
166	84.0881	46.8959	1084.7000	2022-07-23

167	84.0876	46.8965	1043.2000	2022-07-23
168	84.0738	46.9191	1183.9000	2022-07-23
169	84.0458	46.9235	1262.7000	2022-07-23
170	84.0458	46.9235	1308.0900	2022-07-23
171	84.1429	46.8275	0.0000	2022-07-23
172	84.1429	46.8275	0.0000	2022-07-23
173	84.1106	46.9346	1180.2000	2022-07-23
174	84.6986	46.8376	2329.0000	2022-07-22
175	84.6985	46.8376	2327.4000	2022-07-22
176	84.6985	46.8376	2287.1900	2022-07-22
177	84.6924	46.8516	2273.4000	2022-07-22
178	84.6925	46.8517	2317.5000	2022-07-22
179	84.6924	46.8516	2273.4000	2022-07-22
180	84.6925	46.8516	2315.6000	2022-07-22
181	84.6683	46.8641	2235.6000	2022-07-22
182	84.6605	46.8659	2153.5000	2022-07-22
183	84.6925	46.8866	2108.6900	2022-07-22
184	84.6925	46.8866	2064.4000	2022-07-22
185	84.6926	46.8866	2112.1000	2022-07-22
186	84.7022	46.9027	2046.7000	2022-07-22
187	84.7021	46.9029	2041.9000	2022-07-22
188	84.7207	46.9132	1977.1000	2022-07-22
189	84.7065	46.9343	1743.1000	2022-07-22
190	84.6615	46.9450	1481.4000	2022-07-22

191	84.6464	46.9589	1420.5000	2022-07-22
192	84.5952	46.9498	1373.7000	2022-07-22
193	84.5953	46.9498	1336.5000	2022-07-22
194	82.9720	47.0077	1006.3000	2022-07-21
195	82.9898	47.0250	1061.2000	2022-07-21
196	82.9963	47.0342	1098.8000	2022-07-21

Table S11 Grassland investigation site information and completion time of the 10th division of Xinjiang Production and Construction Corps

	Longitude	Latitude	Altitude(m)	Completion time
1	86.2652	46.0492	281.5900	2023-06-30
2	86.2756	46.0765	304.1200	2023-06-30
3	86.2668	46.1207	342.0700	2023-06-30
4	86.2810	46.1356	346.3100	2023-06-30
5	86.2942	46.1989	413.1300	2023-06-30
6	86.4057	46.4021	4.5900	2023-06-30
7	85.6193	47.5637	625.8500	2023-06-29
8	85.7127	48.3402	504.4600	2023-06-29
9	87.9743	48.3721	1694.9600	2023-06-28
10	87.8325	48.4288	1784.4300	2023-06-28
11	87.6585	48.0810	1380.3300	2023-06-27
12	87.6779	48.0677	1379.8500	2023-06-27
13	87.9165	47.6984	728.2500	2023-06-27
14	87.9351	47.6593	633.7700	2023-06-27
15	87.9270	47.6465	596.6100	2023-06-27
16	87.8402	47.6565	552.4500	2023-06-27
17	87.8288	47.6616	538.4800	2023-06-27
18	86.3797	47.1347	1466.6200	2023-06-26
19	86.3275	47.1076	1593.0800	2023-06-26
20	86.3158	47.0763	1739.0200	2023-06-26
21	86.4643	46.3726	492.7000	2022-07-14
22	86.4375	46.3749	503.4000	2022-07-14

23	86.2744	46.0775	310.4100	2022-07-14
24	86.2748	46.0773	303.0200	2022-07-14
25	86.2719	46.1128	337.2800	2022-07-14
26	86.3942	46.3703	519.6000	2022-07-14
27	86.2829	46.1386	348.2300	2022-07-14
28	86.2831	46.1396	352.2600	2022-07-14
29	86.2840	46.1718	364.2800	2022-07-14
30	86.4052	46.4018	556.7000	2022-07-14
31	86.3833	46.3857	530.7000	2022-07-14
32	86.3833	46.3857	530.7000	2022-07-14
33	86.3499	46.3434	471.5000	2022-07-14
34	86.4754	46.3428	461.1000	2022-07-14
35	86.5304	46.3192	465.9000	2022-07-14
36	86.4052	46.3454	463.3000	2022-07-14
37	86.2584	46.0513	281.7000	2022-07-14
38	86.2231	46.0961	333.1000	2022-07-14
39	86.1835	46.1100	359.2000	2022-07-14
40	86.1835	46.1100	359.7000	2022-07-14
41	86.1532	46.1228	380.1000	2022-07-14
42	86.2915	46.1962	383.1000	2022-07-14
43	86.2504	46.1709	380.7900	2022-07-14
44	86.2161	46.1508	375.2000	2022-07-14
45	85.6236	47.4998	727.1900	2022-07-13
46	85.7048	48.3337	440.5000	2022-07-12

47	85.6984	48.3103	440.5000	2022-07-12
48	85.6765	48.2632	438.0000	2022-07-12
49	85.6544	48.2320	423.6900	2022-07-12
50	85.5725	48.1380	392.7000	2022-07-12
51	85.5628	48.1265	391.1000	2022-07-12
52	85.5597	48.1238	390.6000	2022-07-12
53	85.5642	48.0936	380.7900	2022-07-12
54	87.9253	48.3976	1670.0000	2022-07-11
55	87.9556	48.3925	1750.5000	2022-07-11
56	87.9460	48.3859	1779.7000	2022-07-11
57	87.9462	48.3858	1778.0000	2022-07-11
58	87.6583	48.0811	1345.0000	2022-07-09
59	87.6417	48.0648	1277.5300	2022-07-09
60	87.6758	48.0691	1363.5700	2022-07-09
61	87.6838	48.0831	1391.1000	2022-07-09
62	87.8857	47.6729	688.6000	2022-07-08
63	87.8857	47.6729	688.6000	2022-07-08
64	87.9174	47.6261	542.0000	2022-07-08
65	87.9268	47.6452	592.1000	2022-07-08
66	86.3817	47.1348	1454.3000	2022-07-07
67	86.3264	47.1089	1573.9000	2022-07-07

Table S12 Grassland investigation site information and completion time of the 12th division of Xinjiang Production and Construction Corps

	Longitude	Latitude	Altitude(m)	Completion time
1	85.2454	43.0843	2608.5900	2023-07-27
2	85.2606	43.0849	2595.2000	2023-07-27
3	85.3148	43.0915	2597.1200	2023-07-27
4	85.3949	43.1013	2673.7100	2023-07-27
5	85.4523	43.0892	2733.4000	2023-07-27
6	85.4223	43.0610	2657.8900	2023-07-27
7	86.5197	42.9302	2414.5400	2023-07-26
8	86.5381	42.9481	2509.4800	2023-07-26
9	86.5428	42.9649	2559.7500	2023-07-26
10	86.5428	42.9649	2561.2300	2023-07-26
11	86.5818	42.9488	2655.9700	2023-07-26
12	86.6160	42.9362	2751.3200	2023-07-26
13	86.6160	42.9362	2751.6300	2023-07-26
14	86.7493	43.1001	3671.6700	2023-07-26
15	86.7311	43.1709	3075.0900	2023-07-26
16	86.7352	43.0852	3463.4900	2023-07-26
17	86.7343	43.0651	3402.5800	2023-07-26
18	86.7381	43.0312	3281.6600	2023-07-26
19	86.7978	43.0718	3460.3100	2023-07-26
20	86.7909	43.0657	3379.8400	2023-07-26
21	86.7840	43.0325	3176.0700	2023-07-26
22	86.7501	43.0134	3071.6200	2023-07-26

23	87.2913	43.0768	2732.3900	2023-07-25
24	87.3101	43.0706	2651.3700	2023-07-25
25	87.3446	43.0569	2475.8300	2023-07-25
26	87.4033	43.0387	2300.3000	2023-07-25
27	87.4345	43.0326	2170.9700	2023-07-25
28	87.4956	43.0095	1917.6900	2023-07-25
29	87.5712	42.9819	1652.0400	2023-07-25
30	88.3948	43.6258	2216.5500	2023-07-24
31	88.3833	43.5970	1945.7500	2023-07-24
32	88.3693	43.5723	1767.3400	2023-07-24
33	87.1877	43.7980	1052.3000	2023-07-23
34	87.1703	43.8136	1095.6500	2023-07-23
35	87.2622	43.8135	885.3400	2023-07-23
36	86.9474	43.7319	1054.9400	2023-07-23
37	86.9694	43.7415	1046.0000	2023-07-23
38	87.0358	43.7327	1187.2700	2023-07-23
39	87.1041	43.7256	1236.7800	2023-07-23
40	87.1566	43.7275	1083.6000	2023-07-23
41	87.2069	43.7278	969.4800	2022-07-23
42	87.2218	43.6315	1235.7200	2022-07-23
43	87.2277	43.6201	1285.2400	2022-07-23
44	87.2453	43.5898	1384.8000	2022-07-23
45	87.4415	43.5990	1287.0500	2022-07-23
46	87.4201	43.6074	1295.6800	2022-07-23

47	87.2371	43.2525	2489.3900	2022-07-22
48	87.2382	43.2530	2495.3900	2022-07-22
49	87.2408	43.2531	2487.6000	2022-07-22
50	87.2505	43.2503	2479.6900	2022-07-22
51	87.2522	43.2507	2479.6900	2022-07-22
52	87.2269	43.2532	2492.3900	2022-07-22
53	86.7084	43.4379	2095.9000	2022-07-22
54	86.7423	43.4207	1974.3800	2022-07-22
55	86.7471	43.4358	1868.2400	2022-07-22
56	86.7472	43.4357	1868.3900	2022-07-21
57	86.7398	43.4857	1581.7800	2022-07-21
58	86.7325	43.5108	1435.5400	2022-07-21
59	87.0322	43.6671	1367.0900	2022-07-21
60	87.0529	43.6703	1435.2900	2022-07-21
61	87.0563	43.6710	1448.4900	2022-07-21
62	87.0552	43.6664	1432.7100	2022-07-21

Table S13 Grassland investigation site information and completion time of the 13th division of Xinjiang Production and Construction Corps

	Longitude	Latitude	Altitude(m)	Completion time
1	92.4514	44.1115	1698.6495	2024-07-27
2	92.3906	44.1333	1683.7951	2024-07-27
3	92.3153	44.1434	1645.4575	2024-07-27
4	92.2873	44.1706	1591.6938	2024-07-27
5	92.2478	44.1399	1634.3337	2024-07-27
6	92.2224	44.1012	1610.7600	2024-07-27
7	92.2518	44.0732	1718.5302	2024-07-27
8	92.2779	44.0535	1749.6175	2024-07-27
9	92.3047	44.0348	1779.3802	2024-07-27
10	92.3433	44.0089	1780.3100	2024-07-27
11	92.3789	43.9734	1833.3228	2024-07-27
12	92.4352	43.9446	1863.5905	2024-07-27
13	92.4773	43.9297	1894.6969	2024-07-27
14	92.5289	43.9010	1950.4334	2024-07-27
15	92.5649	43.8923	1925.1861	2024-07-27
16	92.6082	43.9139	1851.5533	2024-07-22
17	92.6604	43.9203	1794.1981	2024-07-22
18	92.7030	43.8960	1817.3000	2024-07-22
19	92.7430	43.8714	1804.1943	2024-07-22
20	92.7485	43.8525	1803.3694	2024-07-22
21	92.8039	43.8424	1726.9043	2024-07-22

22	92.8596	43.8502	1653.0503	2024-07-22
23	93.1093	43.6448	1614.5494	2024-07-22
24	93.0452	43.6402	1600.0435	2024-07-22
25	92.9833	43.6616	1605.0775	2024-07-20
26	92.9609	43.8965	1661.3596	2024-07-20
27	92.9272	43.9065	1619.3902	2024-07-20
28	92.9128	43.8735	1630.1913	2024-07-20
29	92.8563	43.8880	1683.0836	2024-07-20
30	92.5476	44.0684	1698.5477	2024-07-20
31	92.5933	44.0385	1679.3236	2024-07-20
32	92.6603	43.9969	1681.7623	2024-07-20
33	92.7069	43.9574	1724.8611	2024-07-20
34	92.7535	43.9209	1778.6606	2024-07-20
35	92.7848	43.9011	1727.2108	2024-07-20
36	92.8837	43.8418	1630.7974	2023-07-26
37	93.0217	43.8779	1710.8783	2023-07-26
38	93.0628	43.8941	1777.3243	2023-07-26
39	93.0645	43.8624	1784.4195	2023-07-26
40	93.0825	43.8259	1751.7692	2023-07-26
41	93.0373	43.8033	1675.5652	2023-07-26
42	93.0223	43.8143	1667.1405	2023-07-26
43	92.9743	43.8039	1618.0789	2023-07-26
44	92.9744	43.8039	1617.8772	2023-07-26
45	93.2719	43.5662	1773.9330	2023-07-25

46	93.2678	43.5486	1853.0052	2023-07-25
47	93.3217	43.5296	2024.8323	2023-07-25
48	93.3316	43.5746	1772.1865	2023-07-25
49	93.6315	43.5852	2090.9275	2023-07-24
50	93.6048	43.5983	2087.7277	2023-07-24
51	93.5715	43.5875	1947.3000	2023-07-24
52	93.5630	43.6012	2036.3776	2023-07-24
53	93.5892	43.6179	2118.1747	2023-07-24
54	93.5645	43.6336	2070.2570	2023-07-24
55	93.5559	43.6461	2107.3721	2023-07-24
56	93.5416	43.6556	2124.8129	2023-07-24
57	93.5419	43.6687	2206.3948	2023-07-24
58	94.3031	43.0126	3461.0000	2023-07-23
59	94.2798	43.0098	3592.7709	2023-07-23
60	94.2456	43.0120	3262.6691	2023-07-23
61	94.2560	42.9745	3188.2265	2023-07-23
62	94.2560	42.9838	2944.8824	2023-07-23
63	94.1768	42.9825	2375.7153	2023-07-23
64	94.1658	42.9825	2227.0767	2023-07-23
65	92.6759	43.1934	1211.5649	2023-07-22
66	92.6363	43.2124	1246.5284	2023-07-22
67	92.5992	43.2363	1283.8669	2023-07-22
68	92.5264	43.2568	1330.9198	2023-07-22
69	92.4227	43.2566	1304.5417	2023-07-22

70	92.3617	43.2598	1304.7361	2023-07-22
71	92.3290	43.2617	1283.9638	2023-07-22
72	92.3139	43.2900	1372.4544	2023-07-22
73	92.3199	43.3253	1502.9748	2023-07-22
74	92.3199	43.3253	1502.8753	2023-07-22
75	92.8791	43.1048	959.3140	2023-07-22
76	92.8967	43.1510	1079.0069	2023-07-22
77	92.9188	43.2189	1262.9400	2023-07-22
78	92.9324	43.2558	1467.7270	2023-07-22
79	92.9411	43.2828	1582.5488	2023-07-22
80	94.7615	42.8962	2145.5103	2023-07-21
81	94.7769	42.9102	2241.9428	2023-07-21
82	94.8095	42.9287	2328.4008	2023-07-21
83	94.8434	42.9532	2515.1204	2023-07-21
84	94.8850	42.9665	2280.0410	2023-07-21
85	94.9112	42.9891	2230.7637	2023-07-21
86	94.9001	42.9747	2229.0284	2023-07-21
87	94.8647	42.9842	2336.0851	2023-07-21
88	94.8069	42.9890	2548.9548	2023-07-21
89	94.8055	42.9772	2568.1240	2023-07-21
90	94.8228	42.9824	2480.9742	2023-07-21
91	95.2421	42.0186	1582.7497	2023-07-20
92	95.2836	42.0381	1615.5577	2023-07-20
93	95.3210	42.0641	1633.9295	2023-07-20

94	95.3258	42.0735	1620.2982	2023-07-20
95	95.3031	42.1031	1548.3680	2023-07-20
96	95.3416	42.1099	1565.1905	2023-07-20
97	95.3781	42.1267	1574.1160	2023-07-20
98	95.4141	42.1416	1509.8000	2023-07-20
99	95.4360	42.1615	1568.1980	2023-07-20
100	95.4932	42.1618	1598.8358	2023-07-20
101	95.5263	42.1537	1633.2221	2023-07-20
102	95.5656	42.1452	1692.7453	2023-07-20
103	95.6112	42.1410	1711.2862	2023-07-20

Table S14 Grassland investigation site information and completion time of the 14th division of Xinjiang Production and Construction Corps

	Longitude	Latitude	Altitude(m)	Completion time
1	81.3183	36.1302	3073.2245	2023-08-05
2	81.2735	36.1326	2819.1617	2023-08-05
3	81.2688	36.1785	2630.1012	2023-08-05
4	81.2742	36.2202	2471.4827	2023-08-05
5	81.0809	36.0921	3123.1115	2023-08-04
6	81.1357	36.0677	3441.4287	2023-08-04
7	81.1222	36.0692	3430.5800	2023-08-04
8	81.1088	36.0737	3330.6045	2023-08-04
9	81.0825	36.0676	3344.5793	2023-08-04
10	81.0637	36.0649	3525.6078	2023-08-04
11	81.0543	36.0726	3449.9468	2023-08-04
12	81.0342	36.0646	3383.4095	2023-08-04
13	81.0153	36.0835	3274.5675	2023-08-04
14	81.0116	36.0978	3218.4125	2023-08-04
15	81.0018	36.0984	2977.8815	2023-08-04
16	80.9904	36.0775	3216.2179	2023-08-04
17	80.9920	36.0683	3080.0000	2023-08-04
18	80.9750	36.0795	2945.1185	2023-08-04
19	80.9629	36.0935	2817.0686	2023-08-04
20	80.9553	36.1010	2775.3149	2023-08-04
21	80.9420	36.1143	2654.9100	2023-08-04
22	80.9370	36.1250	2584.2826	2023-08-04

23	80.9464	36.1406	2534.3252	2023-08-04
24	80.9487	36.1535	2599.2400	2023-08-04
25	80.9275	36.1467	2848.8701	2023-08-04
26	80.9211	36.1679	2747.4782	2023-08-04
27	80.9069	36.1929	3189.1986	2023-08-04
28	80.9069	36.1931	3165.5000	2023-08-04
29	80.8810	36.1701	3279.8474	2023-08-04
30	80.8755	36.1597	3267.3283	2023-08-04
31	80.8410	36.1505	3292.1390	2023-08-04
32	80.8319	36.1634	3231.4697	2023-08-04
33	80.8344	36.1816	3148.9521	2023-08-04
34	80.8296	36.1989	3043.9110	2023-08-04
35	80.8307	36.2102	2926.8409	2023-08-04
36	80.8278	36.2224	2779.1337	2023-08-04
37	80.8281	36.2299	2712.8397	2023-08-04
38	80.8083	36.2657	2443.8505	2023-08-04
39	80.7983	36.2519	2541.2992	2023-08-04
40	80.7827	36.2408	2679.1436	2023-08-04
41	80.7611	36.2336	2810.7100	2023-08-04
42	80.7313	36.2065	3017.5562	2023-08-04
43	80.7227	36.1902	3113.4717	2023-08-04
44	80.7424	36.1817	3138.3165	2023-08-04
45	81.3169	36.1308	3074.5900	2023-07-06
46	81.3173	36.1306	3139.1620	2023-07-06

47	81.2734	36.1325	2891.3422	2023-07-06
48	81.2686	36.1749	2719.9630	2023-07-06
49	81.2684	36.1749	2651.6200	2023-07-06
50	81.2741	36.2203	2541.4118	2023-07-06
51	81.0841	36.0874	3102.8100	2023-07-06
52	81.1356	36.0678	3419.7900	2023-07-06
53	81.1238	36.0685	3422.6200	2023-07-06
54	81.1233	36.0687	3424.3400	2023-07-06
55	81.1087	36.0736	3310.9900	2023-07-06
56	81.0814	36.0647	3351.1800	2023-07-06
57	81.0579	36.0700	3546.1190	2023-07-06
58	81.0338	36.0643	3365.3600	2023-07-06
59	81.0158	36.0812	3266.3400	2023-07-06
60	81.0117	36.0978	3203.5300	2023-07-05
61	81.0013	36.0987	2936.6300	2023-07-05
62	80.9904	36.0780	3205.7800	2023-07-05
63	80.9915	36.0694	3181.9109	2023-07-05
64	80.9915	36.0694	3101.0800	2023-07-05
65	80.9914	36.0694	3099.7300	2023-07-05
66	80.9742	36.0803	2899.3400	2023-07-05
67	80.9635	36.0932	2797.4600	2023-07-05
68	80.9550	36.1014	2753.0500	2023-07-05
69	80.9428	36.1143	2669.2600	2023-07-05
70	80.9369	36.1249	2633.8548	2023-07-05

71	80.9368	36.1249	2542.6600	2023-07-05
72	80.9497	36.1450	2540.8600	2023-07-05
73	80.9492	36.1543	2597.7400	2023-07-05
74	80.9304	36.1446	2809.1100	2023-07-05
75	80.9250	36.1677	2738.3400	2023-07-05
76	80.9097	36.1948	3155.3100	2023-07-05
77	80.9106	36.1947	3150.2000	2023-07-05
78	80.8805	36.1690	3260.0600	2023-07-05
79	80.8773	36.1613	3256.7600	2023-07-05
80	80.8550	36.1457	3299.6700	2023-07-05
81	80.8407	36.1507	3282.0500	2023-07-05
82	80.8322	36.1618	3228.8600	2023-07-05
83	80.8353	36.1799	3134.7500	2023-07-05
84	80.8291	36.1993	3087.7394	2023-07-05
85	80.8291	36.1994	3015.1700	2023-07-05
86	80.8306	36.2088	2927.7700	2023-07-05
87	80.8306	36.2088	2928.1300	2023-07-05
88	80.8278	36.2198	2784.9500	2023-07-05
89	80.8292	36.2277	2700.2300	2023-07-05
90	80.8292	36.2277	2700.2000	2023-07-05
91	80.8292	36.2277	2699.7800	2023-07-05
92	80.8079	36.2654	2429.7000	2023-07-05
93	80.7989	36.2529	2586.3187	2023-07-05
94	80.7987	36.2526	2522.8500	2023-07-05

95	80.7824	36.2406	2719.2733	2023-07-05
96	80.7601	36.2334	2818.8500	2023-07-05
97	80.7312	36.2064	2997.4400	2023-07-05
98	80.7229	36.1894	3094.7700	2023-07-05
99	80.7424	36.1820	3118.2400	2023-07-05
100	80.9487	36.1535	2599.2400	2024-08-04
101	80.9275	36.1467	2848.8701	2024-08-04
102	80.9211	36.1679	2747.4782	2024-08-04
103	80.9069	36.1929	3189.1986	2024-08-04
104	80.9069	36.1931	3165.5000	2024-08-04
105	80.8810	36.1701	3279.8474	2024-08-04
106	80.8755	36.1597	3267.3283	2024-08-04
107	80.8410	36.1505	3292.1390	2024-08-04
108	80.8319	36.1634	3231.4697	2024-08-04
109	80.8344	36.1816	3148.9521	2024-08-04
110	80.8296	36.1989	3043.9110	2024-08-04
111	80.8307	36.2102	2926.8409	2024-08-04
112	80.8278	36.2224	2779.1337	2024-08-04
113	80.8281	36.2299	2712.8397	2023-08-04
114	80.8083	36.2657	2443.8505	2024-08-04
115	80.7983	36.2519	2541.2992	2024-08-04
116	80.7827	36.2408	2679.1436	2024-08-04
117	80.7611	36.2336	2810.7100	2024-08-04
118	80.7313	36.2065	3017.5562	2024-08-04

119	80.7227	36.1902	3113.4717	2024-08-04
120	80.7424	36.1817	3138.3165	2024-08-04
121	81.3169	36.1308	3074.5900	2024-07-06
122	81.3173	36.1306	3139.1620	2024-07-06
123	81.2734	36.1325	2891.3422	2024-07-06
124	81.2686	36.1749	2719.9630	2024-07-06
125	81.2684	36.1749	2651.6200	2024-07-06
126	81.2741	36.2203	2541.4118	2024-07-06
127	81.0841	36.0874	3102.8100	2024-07-06
128	81.1356	36.0678	3419.7900	2024-07-06
129	81.1238	36.0685	3422.6200	2024-07-06
130	81.1233	36.0687	3424.3400	2024-07-06
131	81.1087	36.0736	3310.9900	2024-07-06
132	81.0814	36.0647	3351.1800	2024-07-06
133	81.0579	36.0700	3546.1190	2024-07-06
134	81.0338	36.0643	3365.3600	2024-07-06
135	81.0158	36.0812	3266.3400	2024-07-06
136	81.0117	36.0978	3203.5300	2024-07-05
137	81.0013	36.0987	2936.6300	2024-07-05
138	80.9904	36.0780	3205.7800	2024-07-05
139	80.9487	36.1535	2599.2400	2024-08-04
140	80.9275	36.1467	2848.8701	2024-08-04
141	80.9211	36.1679	2747.4782	2024-08-04
142	80.9069	36.1929	3189.1986	2024-08-04

143	80.9069	36.1931	3165.5000	2024-08-04
144	80.8810	36.1701	3279.8474	2024-08-04
145	80.8755	36.1597	3267.3283	2024-08-04
